# *Dendrobium huoshanense* C. Z. Tang & S. J. Cheng alleviates atherosclerosis by reducing lipid and improving vascular endothelial dysfunction

**DOI:** 10.3389/fnut.2025.1649161

**Published:** 2025-09-26

**Authors:** Xiang-Cheng Fan, Wei-You Cao, Min-Yang He, Hui-Kai Wang, Qing Hao, Wen-Jing Liu, Zhao-Ying Ren, Li-Jun Wang, Jing-Yu Wang, Fei-Xue Wang, Lin Jiang, Qiu-Sheng Zheng, Jun Ma, Feng Zhang, Ji-Chun Han, Lei Zheng

**Affiliations:** ^1^Department of Pharmacy, Center for Membrane Receptor and Brain Medicine, The Fourth Affiliated Hospital of School of Medicine, and International School of Medicine, International Institutes of Medicine, Zhejiang University, Yiwu, China; ^2^Department of Cardiovascular Surgery, Yantai Yuhuangding Hospital, Yantai, China; ^3^College of Traditional Chinese Medicine, Binzhou Medical University, Yantai, China; ^4^Department of Gastroenterology, The Fourth Affiliated Hospital of School of Medicine, and International School of Medicine, International Institutes of Medicine, Zhejiang University, Yiwu, China; ^5^Binzhou Medical University Affiliated Traditional Chinese Medicine Hospital, Binzhou Medical University, Binzhou, China; ^6^Department of Pharmacy, Changzheng Hospital, Naval Medical University, Shanghai, China

**Keywords:** *Dendrobium huoshanense* C. Z. Tang & S. J. Cheng, atherosclerosis, homology-of-medicine-and-food, functional food, zebrafish

## Abstract

**Introduction:**

*Dendrobium huoshanense* C. Z. Tang & S. J. Cheng (DH) water extract has previously been found to have therapeutic effects on atherosclerosis (AS), but the active ingredients responsible for these effects remain unclear. This study aims to identify the active monomers in DH water extract that can treat AS, providing a basis for its use in preventing AS.

**Methods:**

LC-MS analysis was conducted to identify the main active ingredients in DH water extract. A zebrafish AS model was used to screen for the active ingredients with therapeutic effects. Network pharmacology was utilized to predict the mechanisms through which these active ingredients treat AS.

**Results:**

Six main active ingredients were identified, and three, namely dendrophenol, naringenin, and apigenin-G, were selected for further study. Network pharmacology analysis indicated that naringenin and apigenin-G may treat AS by lowering blood lipid levels, including total cholesterol, triglycerides, low-density lipoprotein (LDL), and very low-density lipoprotein (VLDL), while dendrophenol may improve low shear stress (LSS)-induced endothelial cell dysfunction. Zebrafish model studies showed that naringenin and apigenin-G significantly reduced cholesterol, triglycerides, LDL, and VLDL levels. In the LSS-induced endothelial cell dysfunction model, dendrophenol significantly increased nitric oxide (NO) activity and reduced reactive oxygen species (ROS) activity.

**Discussion:**

The findings suggest that DH may treat AS through the lipid-lowering effects of naringenin and apigenin-G, and by improving endothelial cell dysfunction via dendrophenol. Given that DH is a homology-of-medicine-and-food, it is suitable for preparation as a functional food aimed at preventing AS.

## 1 Introduction

Atherosclerosis (AS) is the basic disease of almost all cardiovascular diseases, especially the main cause of coronary heart disease, cerebral infarction and other vascular diseases with high mortality. Approximately 2 billion people worldwide suffer from AS. In China, the number of AS patients is as high as 270 million, equivalent to more than one-fifth of the total population (1, 2). Once AS is formed, existing treatment methods cannot completely cure it. Only risk factors such as statin lipid-lowering drugs, hypoglycemic drugs, and antihypertensive drugs can be controlled to make the plaque relatively stable or progress slowly. This not only poses a serious threat to the physical and mental health of patients, but also increases their economic burden. Therefore, finding effective methods to prevent the occurrence of AS is an urgent task that needs to be addressed.

The pathogenesis of AS is complex, with multiple mechanisms involved in its occurrence, among which hyperlipidemia is considered one of the main risk factors (3, 4). Elevated lipid levels in the blood can directly cause lipid accumulation on the inner walls of blood vessels and develop into plaques (5). The most common medication currently used to treat AS is also lipid-lowering drugs, such as statins. These lipid-lowering drugs have a good improvement effect on the aggravation and stability of plaques, but cannot completely cure AS (6, 7). This may also be because the pathogenesis of AS is complex, and single lipid-lowering therapy is difficult to cure AS. Although lipid-lowering cannot completely cure AS, it has a good effect on preventing and delaying AS. Therefore, finding foods or drugs with lipid-lowering effects to prevent and treat AS is a feasible approach.

Low shear stress (LSS) is also considered one of the important factors causing AS (8). Unlike hyperlipidemia, LSS is a human factor and not a disease factor. AS plaques are more common in the LSS area of blood vessels, mainly located on the inner side of the vessel curvature and the outer side of the vessel bifurcation (9). LSS can induce a decrease in nitric oxide (NO) activity in endothelial cells, causing dysfunction of endothelial cells and ultimately leading to atherosclerosis. LSS can also induce an increase in reactive oxygen species (ROS) activity in endothelial cells, causing oxidative damage to the vascular endothelium and ultimately developing into AS (10, 11). These findings highlight the importance of vascular biomechanics in AS pathogenesis and suggest that targeting LSS-induced endothelial dysfunction could be a promising strategy for AS prevention and treatment.

*Dendrobium huoshanense* C. Z. Tang & S. J. Cheng (DH) is a traditional Chinese medicine and also a food, belonging to the same origin of medicine and food, with anti-inflammatory and antioxidant effects (12). Our previous research has found that DH has therapeutic effects on AS, and DH can not only reduce blood lipids but also improve LSS induced endothelial cell dysfunction (13). The identification of these active compounds is crucial for understanding DH’s mechanism of action and for developing targeted therapies or functional foods for AS prevention. Therefore, the purpose of this study is to identify the active monomers in DH that are effective against AS, providing a scientific basis for using DH in AS prevention strategies.

## 2 Materials and methods

### 2.1 Chemicals and materials

DH was purchased from Anhui Hushengji Biotechnology Co., Ltd. (Hefei, Anhui Province, China). Dendrophenol, naringenin, and apigenin-G (purity ≥98%) were purchased from Chengdu Must Bio-Technology Co., Ltd. (Chengdu, China). CholEsteryl BODIPY^™^ FL C12 was purchased from Thermo Fisher Scientific (Waltham, MA, United States; Catalog No. C3927MP). Cholesterol (purity ≥98%) was purchased from Beijing Solarbio Science & Technology Co., Ltd. (Beijing, China; Catalog No. IC0370). Dihydroethidium (DHE), and 4-amino-5-aminomethyl-2′,7′-difluorescein diacetate (DAFFM DA) were purchased from Nanjing Beyotime Biotechnology Co., Ltd. (Nanjing, Jiangsu Province, China). Nile Red was purchased from Beijing Solarbio Science & Technology Co., Ltd. (Beijing, China).

### 2.2 LC-MS analysis

Use ACQUITY UPLC I-Class/AB SCIEX X500B series QTOF mass spectrometer with ACQUITY UPLC chromatographic column ® HSS T3 (2.1 × 100 mm, 1.8 μm), mobile phase A is acetonitrile, B is 0.1% formic acid aqueous solution, mobile phase gradient is shown in Table 1, flow rate is 0.3 mL/min, column temperature is 30 °C, injection volume is 3 μL, wavelength is 337 nm. The mass spectrometry conditions are detected separately in positive and negative ion modes using an ESI ion source. Positive ion mode: curtain gas at 35 psi, gas 1: 40 psi, gas 2: 40 psi, temperature 500 °C, ionization pressure 5,000 V, de clustering voltage 70 V, full scanning range m/z 80–1,500, fragmentation voltage 5 V, CE spread 0 V. Negative ion mode: curtain air at 35 psi, gas 1: 40 psi, gas 2: 40 psi, temperature 450 °C, ionization pressure −4,500 V, de clustering voltage −70 V, full scan range m/z 80–1,500, fragmentation voltage −5 V, CE spread 0 V.

**Table 1:** Chromatographic mobile phase gradient.

Time (min)	Mobile phase	
	A% (acetonitrile)	B% (0.1% formic acid)
0	3	97
3	5	95
8	18	82
15	30	70
20	50	50
23	65	35
30	90	10

### 2.3 Zebrafish AS model

According to our research, we adopted a high cholesterol diet (HCD) to establish a zebrafish AS model. The 5-day old wild-type AB-line zebrafish larvae were fed an HCD enriched with 4% cholesterol for 45 days. After feeding HCD for 45 days, plaques will form in the blood vessels of zebrafish, resulting in vascular stenosis. All experimental procedures involving animals were approved by the Medical Ethics Committee of Binzhou Medical University (Approval No. 2024-031). For euthanasia, zebrafish were anesthetized using 150 mg/L tricaine (MS-222, Catalog: A5040, Sigma) prior to euthanasia. Anesthesia was administered for 10 min until the fish exhibited signs of sedation, including the cessation of movement and a lack of response to gentle stimuli. After euthanasia, the zebrafish were fixed in 4% paraformaldehyde for histological analysis. The dosage of tricaine was optimized to minimize distress and ensure humane treatment in accordance with institutional guidelines.

### 2.4 Detection of the effect of vicenin-1, schaftoside, apigenin-G, isoschaftoside, naringenin, and dendrophenol on cholesterol accumulation in AS zebrafish

The 5-day old Tg(*fli1a*:EGFP) (endothelial EGFP) zebrafish larvae were randomly divided into eight groups (*n* = 6 per group): control group, AS group, vicenin-1 group, schaftoside group, apigenin-G group, isoschaftoside group, naringenin group, and dendrophenol group. This comprehensive grouping allows for a thorough evaluation of multiple compounds on atherosclerosis development. In the control group, 5-day old Tg(*fli1a*:EGFP) zebrafish larvae were fed only with 10 μg/g of red fluorescent lipid (without 4% cholesterol) for 10 days. In the AS group, 5-day old Tg(*fli1a*:EGFP) zebrafish larvae were fed an HCD enriched with 4% cholesterol and supplemented with 10 μg/g of red fluorescent lipid for 10 days. In the vicenin-1 group: 5-day old Tg(*fli1a*:EGFP) zebrafish larvae were fed an HCD enriched with 4% cholesterol and supplemented with 10 μg/g of red fluorescent lipid for 10 days and treated with 10 mg/L vicenin-1. In the schaftoside group: 5-day old Tg(*fli1a*:EGFP) zebrafish larvae were fed an HCD enriched with 4% cholesterol and supplemented with 10 μg/g of red fluorescent lipid for 10 days and treated with 10 mg/L schaftoside. In the apigenin-G group: 5-day old Tg(*fli1a*:EGFP) zebrafish larvae were fed an HCD enriched with 4% cholesterol and supplemented with 10 μg/g of red fluorescent lipid for 10 days and treated with 10 mg/L apigenin-G. In the isoschaftoside group: 5-day old Tg(*fli1a*:EGFP) zebrafish larvae were fed an HCD enriched with 4% cholesterol and supplemented with 10 μg/g of red fluorescent lipid for 10 days and treated with 10 mg/L isoschaftoside. In the naringenin group: 5-day old Tg(*fli1a*:EGFP) zebrafish larvae were fed an HCD enriched with 4% cholesterol and supplemented with 10 μg/g of red fluorescent lipid for 10 days and treated with 10 mg/L naringenin. In the dendrophenol group: 5-day old Tg(*fli1a*:EGFP) zebrafish larvae were fed an HCD enriched with 4% cholesterol and supplemented with 10 μg/g of red fluorescent lipid for 10 days and treated with 10 mg/L dendrophenol. Images of the caudal vasculature in live larvae show diffuse red fluorescence of circulating fluorescent lipids in both the control and HCD-fed larvae and bright fluorescent lipid deposits in the blood vessel wall only in HCD-fed larvae. Studies have found that these accumulated lipids are similar to the plaques of early AS. The research results showed that dendrophenol, naringenin, and apigenin-G can significantly reduce cholesterol accumulation in the blood vessels of AS zebrafish. Based on these findings, these three compounds were selected for further investigation.

### 2.5 Evaluation of dendrophenol, naringenin, and apigenin-G effects on neutrophil infiltration in AS zebrafish

Five-day-old *Tg*(*mpx:EGFP*) zebrafish larvae, which express GFP in neutrophils, were randomly divided into five groups (*n* = 6 per group): control, AS, dendrophenol, naringenin, and apigenin-G. In the control group, zebrafish larvae were fed only normal basal feed (without 4% cholesterol) for 10 days. In the AS group, zebrafish larvae were fed an HCD enriched with 4% cholesterol for 10 days (25). In the treatment groups (dendrophenol, naringenin, and apigenin-G), zebrafish larvae were fed an HCD enriched with 4% cholesterol for 10 days and simultaneously treated with 10 mg/L of the respective compound. After 10 days of feeding and a subsequent 24-h fasting period, the number of neutrophils was observed under a fluorescence microscope. This fasting period ensures that any observed effects are due to long-term changes rather than acute feeding responses.

### 2.6 Assessment of dendrophenol, naringenin, and apigenin-G effects on macrophage infiltration in AS zebrafish

Five-day-old Tg(lyz:DsRED2) zebrafish larvae, which express red fluorescent protein in macrophages, were randomly divided into five groups (*n* = 6 per group): control, AS, dendrophenol, naringenin, and apigenin-G. The feeding and treatment protocols were identical to those described in section 2.5. This consistency allows for direct comparison between neutrophil and macrophage infiltration studies. Macrophages marked with red fluorescence in blood vessels were directly observed under a fluorescence microscope. This direct visualization allows for quantification of macrophage infiltration in the vessel wall.

### 2.7 Evaluation of dendrophenol, naringenin, and apigenin-G effects on plaque formation in AS zebrafish

Five-day-old wild-type AB-line zebrafish larvae were randomly divided into five groups (*n* = 6 per group): control, AS, dendrophenol, naringenin, and apigenin-G. The use of wild-type zebrafish allows for assessment of plaque formation without potential confounding effects from transgenic modifications. In the control group, zebrafish larvae were fed normal basic feed for 45 days (26). In the AS group, zebrafish larvae were fed an HCD enriched with 4% cholesterol for 45 days. In the treatment groups (dendrophenol, naringenin, and apigenin-G), zebrafish larvae were fed an HCD enriched with 4% cholesterol for 45 days and simultaneously treated with 10 mg/L of the respective compound. After 45 days of feeding, the zebrafish were euthanized and fixed with paraformaldehyde. The blood vessels of each group of zebrafish were then analyzed for plaque formation using three complementary staining techniques: H&E, Verhoeff-Van Gieson (EVG), and Oil Red O staining.

### 2.8 Network pharmacology analysis

AS-related disease targets were collected from well-established databases such as GeneCards,^1^ OMIM,^2^ and DisGeNET,^3^ which provide information on genes associated with AS. The structures of the active compounds were obtained from PubChem, and their corresponding SMILES representations were used for further analysis. Potential targets for each compound were predicted using the SwissTargetPrediction database,^4^ SEA,^5^ and SuperPred, all of which employ computational methods to predict molecular targets based on compound structures. The intersection of the disease-related targets and the compound targets was visualized using Venn diagrams, created with the R language VennDiagram package. A compound-target network (C-T network) was then constructed using Cytoscape v3.9.0 software to identify and analyze the key biological pathways that may be affected by dendrophenol, naringenin, and apigenin-G. To assess the importance of the nodes within the network, centrality measures such as “betweenness centrality,” “closeness centrality,” and “degree centrality” were calculated. In addition to the compound-target network analysis, we employed the KEGG pathway analysis using Metscape,^6^ which allows for the identification of enriched biological pathways. The following parameters were set: a minimum enrichment value of 1.5, a minimum overlap value of 3, and a *p*-value cutoff of 0.01. It is important to note that these results are primarily based on computational predictions and should be interpreted with caution. They serve as preliminary insights into potential therapeutic mechanisms and will require further experimental validation to confirm their relevance. Finally, the results of the pathway enrichment analysis were exported for further exploration and visualized using SRplot,^7^ an online tool designed for data visualization and analysis.

### 2.9 Molecular docking screening

The 3D structure of protein was downloaded from the Protein Data Bank.^8^ The 3D structures of dendrophenol, naringenin, and apigenin-G were constructed using ChemOffice and PyMOL (version 8.0). Molecular docking was performed using AutoDockTools (version 1.5.7), with ligands docked into a well-defined cavity in the protein crystal structure. A genetic algorithm was applied to explore ligand conformations, with all single bonds of the ligands designated as rotatable. The docking box was centered on the binding region and large enough to cover the entire site, while the protein structure was fixed during docking. Binding affinities were evaluated based on docking scores. Additionally, the molecular docking model was further analyzed using Molecular Operating Environment (MOE 2015, Chemical Computing Group ULC).

### 2.10 Evaluation of naringenin and apigenin-G effects on lipid levels in AS zebrafish

Five-day-old wild-type AB-line zebrafish larvae were randomly divided into four groups (*n* = 6 per group): control, AS, naringenin, and apigenin-G. The sample size was determined based on previous studies showing this provides sufficient statistical power to detect treatment effects. Control group zebrafish were fed a normal basal diet without added cholesterol for 10 days. AS group zebrafish were fed a HCD enriched with 4% cholesterol for 10 days. This HCD model has been previously established to induce atherosclerotic changes in zebrafish larvae. Naringenin group: zebrafish were fed the HCD for 10 days and treated with 10 mg/L naringenin. Apigenin-G group: zebrafish were fed the HCD for 10 days and treated with 10 mg/L apigenin-G. The 10 mg/L dose was selected based on preliminary dose-finding experiments. After 10 days of feeding and a 24-h fasting period, lipid levels were assessed in each group using Nile Red staining. The 24-h fast was implemented to standardize lipid measurements across groups. A stock solution of Nile Red (1.25 mg/mL; Invitrogen N-1142) was prepared in acetone and stored at 20 °C in the dark. For staining, the stock was diluted to 50 ng/mL in egg water and larvae were incubated for 15 min at 28 °C in the dark. These staining conditions were optimized to provide clear visualization of lipids while minimizing background fluorescence. Larvae were washed three times with distilled water and anesthetized with tricaine (Sigma; 4 mg/mL, pH 7.0). Specimens were mounted in 4% methylcellulose and imaged using an OLYMPUS SZX16 microscope (Olympus Corporation, Japan) with yellow fluorescence filters. Multiple larvae per group were imaged to ensure representative results.

### 2.11 Cell culture

The human umbilical vein endothelial cell (HUVEC) line EA.hy926 cells (American Type Culture Collection) were cultured in DMEM (Invitrogen) containing 10% heat-inactivated fetal bovine serum (FBS), penicillin (100 U/mL) and streptomycin (100 μg/mL) at 37 °C in a 5% CO_2_ humid incubator. When the EA.hy926 cells grew to log phase, the cells were seeded onto a glass slide (30 × 50 mm) and treated with DMSO (0.1%) or various concentrations of DH water extract (0.1, 1 and 10 mg/L) for 24 h. After 24 h, the LSS test was started.

### 2.12 Low shear stress

A parallel flow chamber (Shanghai Medical Instrument School, Shanghai, China), which consists of two stainless steel plates and a silicon gasket, was used in this study. A glass slide (30 × 50 mm) with confluent cells was placed on the lower plate of the chamber and then subjected to LSS induced by continuous fluid flow. Shear stress values (3 dyn/cm^2^) were modulated by the flow though the chamber.

### 2.13 Assessment of nitric oxide levels

To determine the intracellular NO level, we incubated EA.hy926 cells with the NO-specific fluorescent dye DAF-FM DA (50 μM, Beyotime Institute of Biotechnology) in culture medium without phenol red at 37 °C for 30 min after treatment with LSS, LSS + dendrophenol (10 mg/L) or not. The EA.hy926 cells were washed in PBS twice after the fluorescent dye treatment and then photographed and analyzed via fluorescence microscopy. Fluorescence intensity was quantified from at least three random fields per slide, with three slides analyzed per condition to ensure reproducibility.

### 2.14 Assessment of reactive oxygen species levels

Intracellular ROS levels were measured using the ROS-specific fluorescent dye dihydroethidium (DHE; 50 μM; Beyotime, China). The staining and imaging protocol was identical to that described for NO assessment. This parallel approach allows for direct comparison between NO and ROS levels under various treatment conditions.

### 2.15 Real-time quantitative PCR (RT-qPCR) analysis

Total RNA was extracted from six larval zebrafish per group at week 2 using TRIzol reagent (Invitrogen, United States). After DNase treatment, RNA quantity and purity were assessed with a NanoDrop spectrophotometer (Thermo Fisher Scientific, United States). cDNA was synthesized from purified RNA using HiScript IV All-in-One Ultra RT SuperMix for qPCR (Catalog No. R433, Vazyme, China). qPCR was performed on a LightCycler 96 real-time PCR system (Roche Biosystems, United States) with ChamQ^™^ Universal SYBR qPCR Master Mix (Vazyme, China). Primer sequences (Sangon Biotech Co., Ltd., Shanghai, China) were listed in Supplementary Table S4. Relative expression was calculated by the 2^−ΔΔCt^ method using GAPDH as the reference gene.

### 2.16 Statistical analysis

Data are presented as the mean ± standard deviation. Statistical differences were determined using analysis of variance (ANOVA), where *p* < 0.05 was considered statistically significant. The analyses were performed using the Statistical Program for Social Sciences Software (IBM SPSS, International Business Machines Corporation, Armonk City, NY, United States).

## 3 Results

### 3.1 Dendrophenol, naringenin, and apigenin-G in DH have therapeutic effects on AS

LC-MS analysis of DH water extract identified six primary active compounds: vicenin-1, schaftoside, apigenin-G, isoschaftoside, naringenin, and dendrophenol. These compounds were characterized based on retention times and mass-to-charge (m/z) ratios, with deviations within 11.82 ppm of theoretical values (Table 2 and Supplementary Figures S1–S12). This comprehensive profiling approach ensures confident identification of the bioactive components in the complex DH extract. Vicenin-1 and schaftoside (m/z 563.1412 and 563.1362, respectively) are known for their antioxidant and anti-inflammatory properties. Apigenin-G (m/z 533.1295) is a flavonoid glycoside linked to lipid-lowering effects. Naringenin (m/z 271.0606) and dendrophenol (m/z 273.1127) have established vascular protective effects, aligning with previous studies on their roles in lipid metabolism and inflammation modulation (14). The identification of these compounds with known beneficial properties supports the potential therapeutic effects of DH extract on AS.

**Table 2:** DH liquid quality identification results.

Number	Retention time (min)	Theoretical value (m/z)	Actual value (m/z)	Error (ppm)	Molecular formula	Component identification
1	8.02	563.1412	563.1401	−1.95	C_26_H_28_O_14_	Vicenin-1
2	8.53	563.1362	563.1401	6.93	C_26_H_28_O_14_	Schaftoside
3	8.96	593.1466	593.1502	6.07	C_27_H_30_O_15_	Chrysoeriol-G
4	9.83	533.1232	533.1295	11.82	C_25_H_26_O_13_	Apigenin-G
5	10.09	563.1342	563.1401	10.48	C_26_H_28_O_14_	Isoschaftoside
6	17.27	271.0584	271.0606	8.12	C_15_H_12_O_5_	Naringenin
7	20.13	273.1106	273.1127	7.69	C_17_H_20_O_5_	Dendrophenol

**Figure 1:**
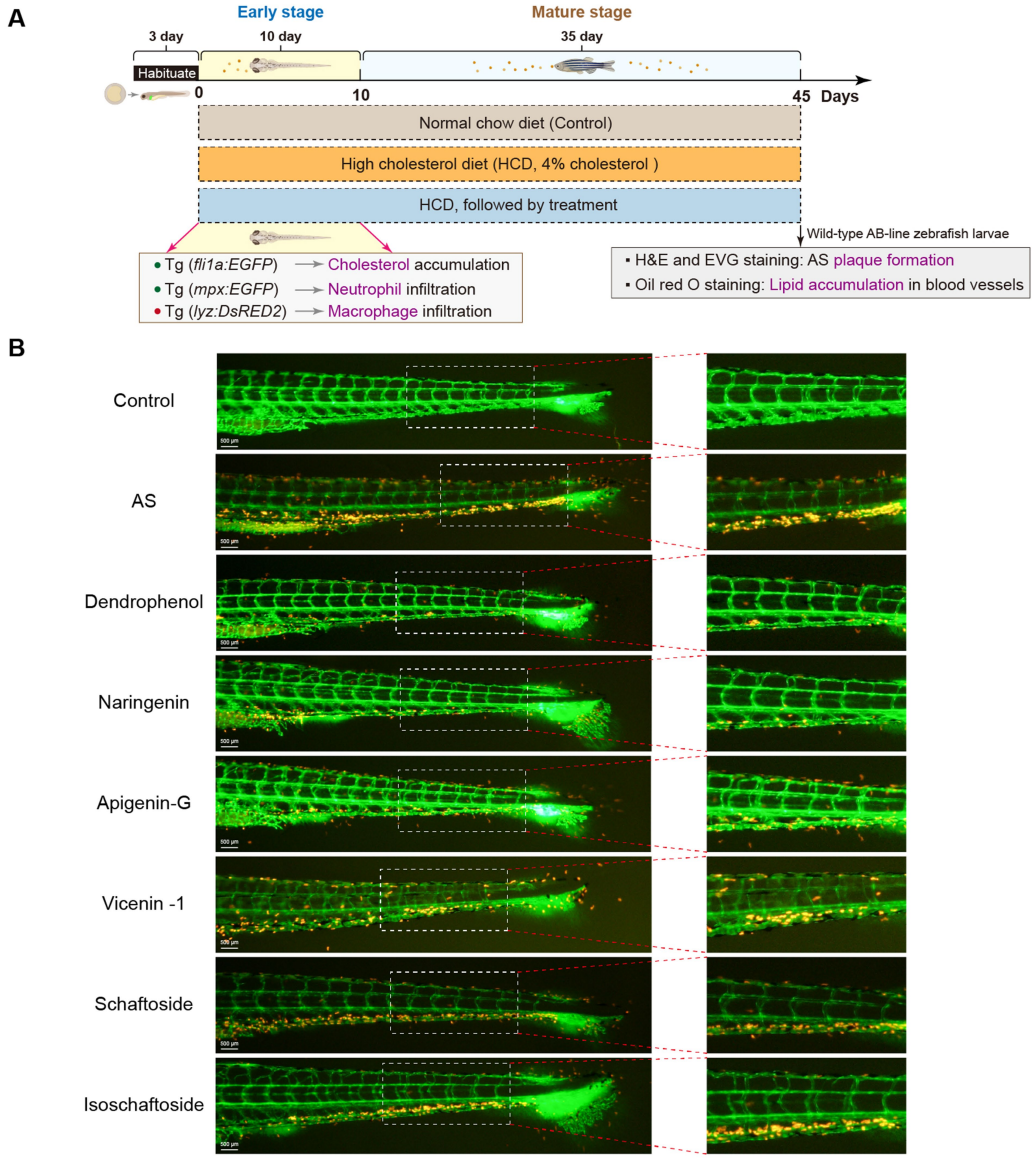
Effects of various compounds on cholesterol accumulation in zebrafish blood vessels. **(A)** Experimental timeline for zebrafish treatment: Zebrafish were first fed a HCD for 45 days to induce AS, followed by treatment with different compounds (Dendrophenol, Naringenin, Apigenin-G, Vicenin-1, Schaftoside, and Isoschaftoside). The diagram shows the experimental setup, highlighting the stages of treatment and key outcomes, including cholesterol accumulation (Tg(*fli1a:EGFP*)) and macrophage infiltration (Tg(*lyz:DsRED2*)) during the early and mature stages of treatment. **(B)** Representative images of zebrafish blood vessels. The left panels show the entire caudal vasculature, with green fluorescence indicating endothelial cells (labeled with Tg(*fli1a:EGFP*)), and red fluorescence indicating cholesterol accumulation. The right panels show magnified views of the boxed areas from the left panels (*n* = 6). Scale bar: 500 μm for the left image.

Based on the LC-MS results, dendrophenol, naringenin, and apigenin-G were selected for further investigation using the zebrafish AS model. These compounds were prioritized due to their previously reported bioactivities relevant to AS pathogenesis. The zebrafish model demonstrated that all three compounds significantly reduced cholesterol accumulation in blood vessels of AS-induced larvae (Figure 1). This *in vivo* evidence supports the hypothesis that these compounds may exert therapeutic effects on AS through lipid-lowering properties.

**Figure 2:**
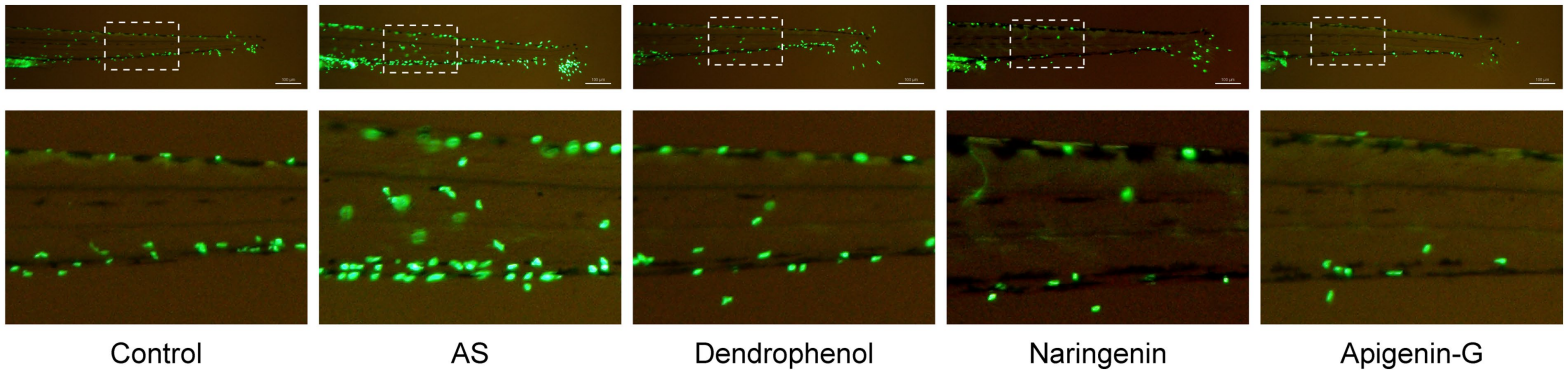
Effects of treatment on neutrophil infiltration in zebrafish blood vessels. The images show neutrophil infiltration (green fluorescence) in the blood vessels of zebrafish subjected to a high-cholesterol diet (AS) and treated with different compounds (dendrophenol, naringenin, and apigenin-G). The above panels show the full caudal vasculature, and the down panels show magnified views of the boxed regions. The neutrophils were labeled using the *Tg(mpx:EGFP)* transgenic zebrafish model. In the AS group, increased neutrophil infiltration was observed, which was reduced following treatment with dendrophenol, naringenin, and apigenin-G (*n* = 6). Scale bar: 100 μm.

**Figure 3:**
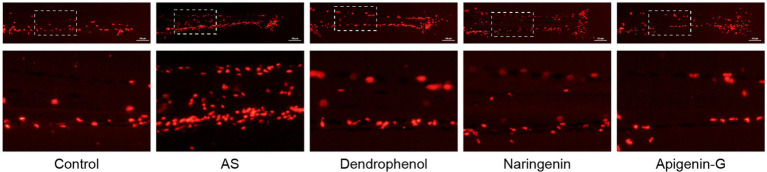
Effects of treatment on macrophage infiltration in zebrafish blood vessels. The images show macrophage infiltration (red fluorescence) in zebrafish blood vessels after high-cholesterol diet (AS) treatment and subsequent treatment with dendrophenol, naringenin, and apigenin-G. The above panels show the full caudal vasculature, and the down panels display magnified views of the boxed regions. Macrophages were labeled using the *Tg(lyz:DsRED2)* transgenic model (*n* = 6). Scale bar: 100 μm.

**Figure 4:**
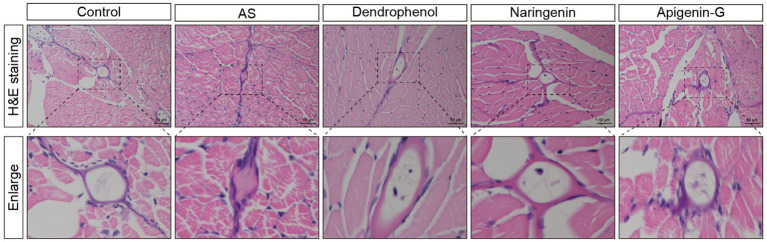
Histological analysis of atherosclerotic plaques in AS zebrafish treated with different compounds. The images show H&E staining of blood vessel sections from control, AS, dendrophenol, naringenin, and apigenin-G groups. The enlarged sections below each image highlight the plaque formation in the blood vessels of the AS zebrafish model. Treatment with dendrophenol, naringenin, and apigenin-G significantly reduced plaque formation compared to the AS group (*n* = 6). Scale bars: 50 μm.

**Figure 5:**
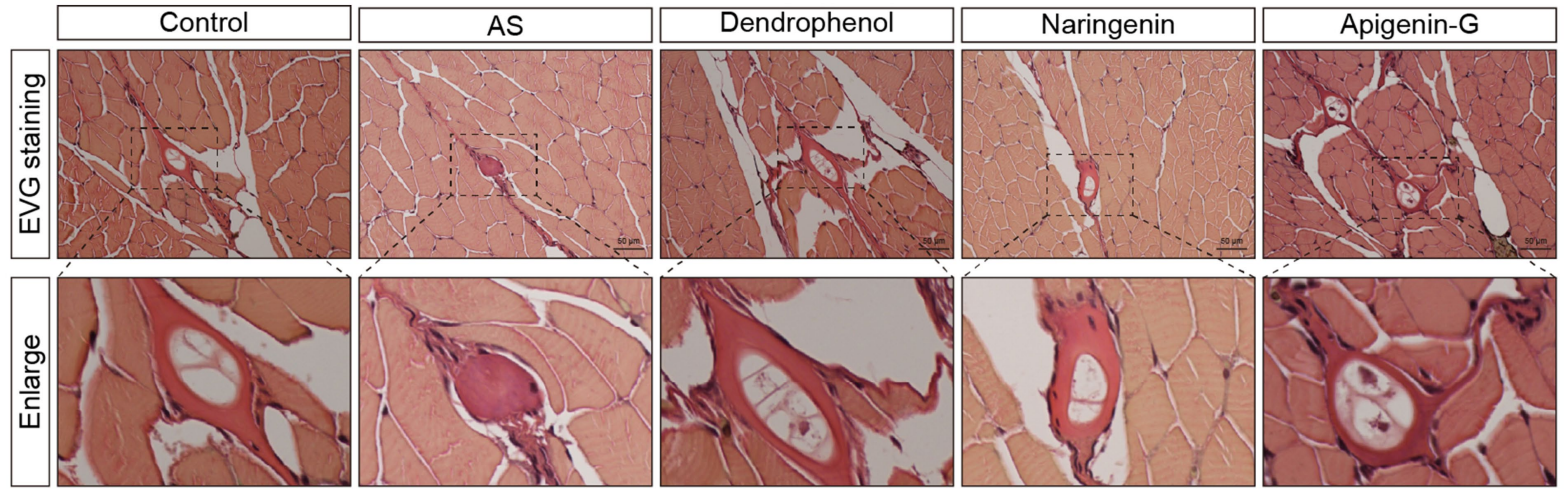
Histological analysis of atherosclerotic plaques in AS zebrafish treated with different compounds using EVG staining. The images show EVG staining of blood vessel sections from control, AS, dendrophenol, naringenin, and apigenin-G groups. The enlarged sections below each image highlight the plaque formation in the blood vessels of the AS zebrafish model (*n* = 6). Scale bars: 50 μm.

**Figure 6:**
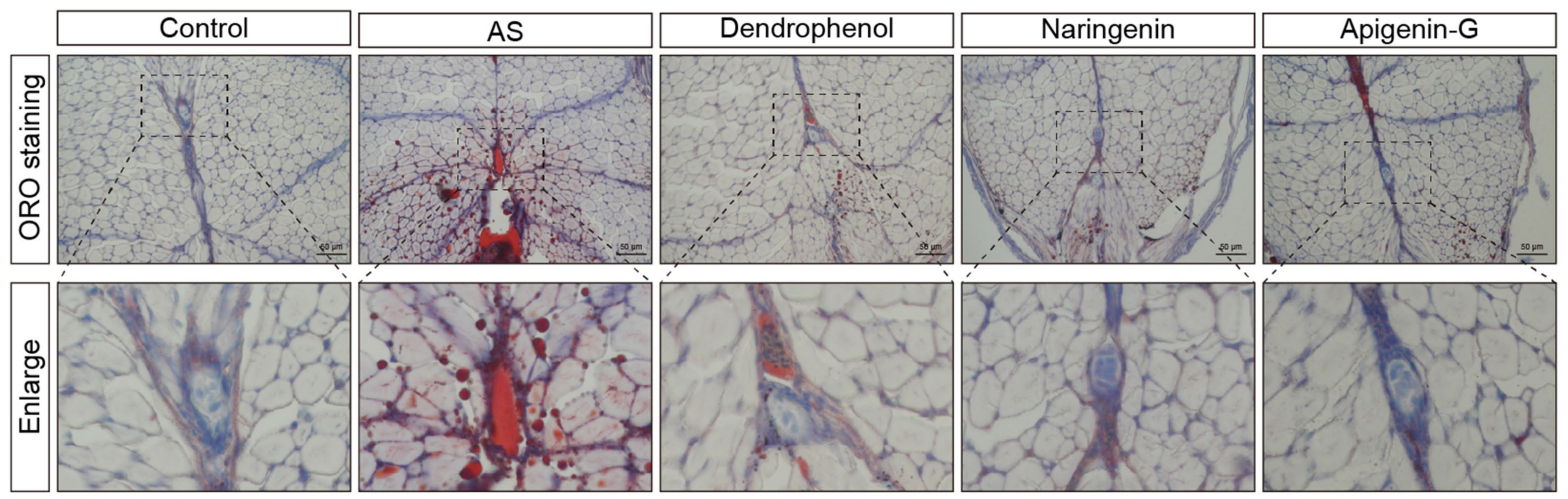
Oil Red O staining analysis of lipid accumulation in blood vessels of AS zebrafish treated with different compounds. The images display Oil Red O staining of blood vessel sections from control, AS, dendrophenol, naringenin, and apigenin-G groups. The enlarged sections below each image highlight lipid deposition in the blood vessels of AS zebrafish. Dendrophenol, naringenin, and apigenin-G treatment led to a significant reduction in lipid accumulation compared to the AS group (*n* = 6). Scale bars: 50 μm.

Further studies revealed that dendrophenol, naringenin, and apigenin-G significantly reduced the accumulation of neutrophils and macrophages in the vascular wall of AS zebrafish and alleviated plaque formation (Figures 2–6). These findings suggest that the compounds may have multifaceted effects on AS, targeting both lipid accumulation and inflammatory processes. Collectively, these results indicate that dendrophenol, naringenin, and apigenin-G in DH water extract may have therapeutic potential for AS treatment.

### 3.2 Apigenin-G may alleviate AS through the “Lipid and atherosclerosis” pathway

**Figure 7:**
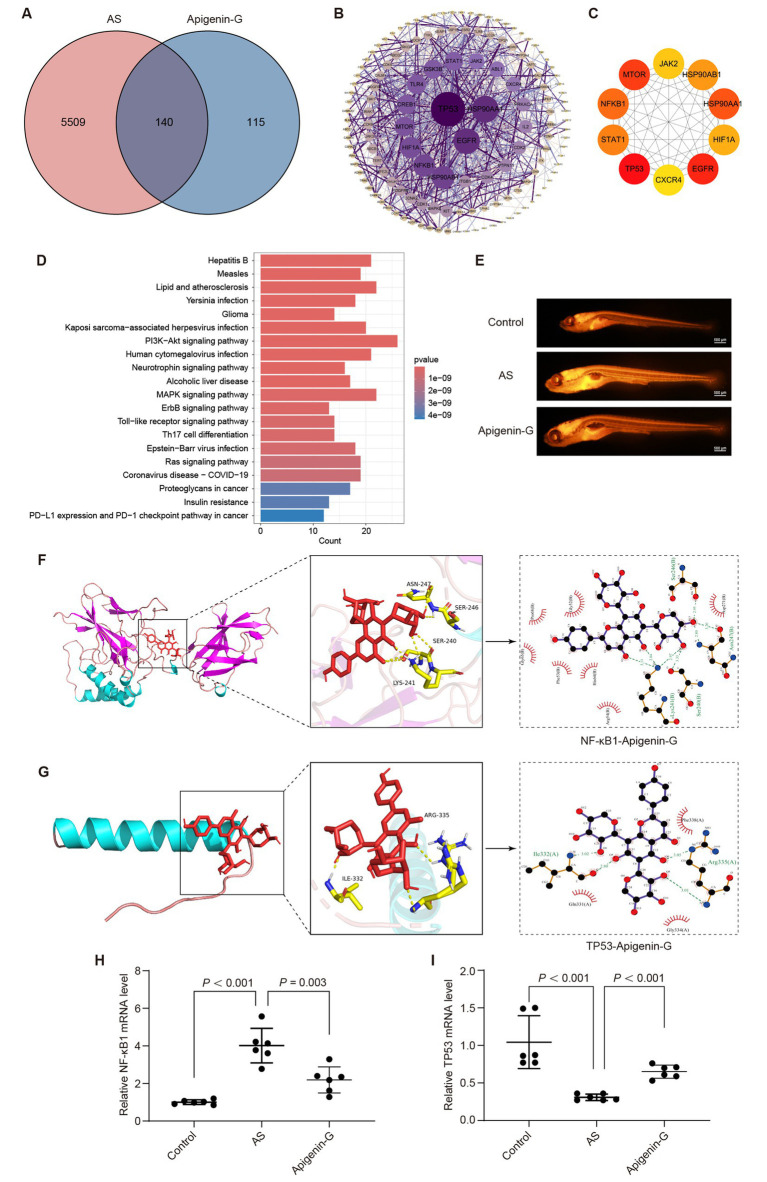
Apigenin-G may alleviate AS through its lipid-lowering effect. **(A)** Venn diagram showing the intersection between apigenin-G-related targets and AS-related targets, identifying 140 common targets for potential therapeutic effects. **(B)** PPI network of apigenin-G with AS-related targets, where darker purple nodes indicate more important proteins. **(C)** The top 10 core targets identified by Cytoscape, including TP53, EGFR, and NF-κB1, which are integral to AS pathology. **(D)** KEGG pathway analysis showing the top 20 pathways, including the “Lipid and atherosclerosis” pathway, where apigenin-G may exert its therapeutic effects. **(E)** Nile Red staining results indicating reduced lipid accumulation in the blood vessels of AS zebrafish treated with apigenin-G. Scale bar = 500 μm. **(F)** Molecular docking analysis showing the binding of apigenin-G to NF-κB1, with a binding energy of −9.7 kcal/mol. **(G)** Molecular docking analysis showing the binding of apigenin-G to TP53, with a binding energy of −9.8 kcal/mol. **(H,I)** qRT-PCR results demonstrating that apigenin-G significantly reduces NF-κB1 mRNA levels **(H)** and restores TP53 mRNA expression **(I)** in zebrafish compared to AS group. Data are presented as mean ± SD (*n* = 6).

Network pharmacology analysis identified 140 intersecting targets between apigenin-G and AS (Figure 7A). This approach provides a systematic method for exploring potential mechanisms of action. Protein–protein interaction (PPI) analysis of these targets was performed using the STRING database (Figure 7B). The PPI network visualization aids in understanding the complex interactions between apigenin-G targets and AS-related proteins.

Cytoscape software with the MCC algorithm identified the top 10 core targets: TP53, EGFR, MTOR, HSP90AA1, NF-κB1, STAT1, HSP90AB1, HIF1A, JAK2, and CXCR4 (Figure 7C). These core targets represent key nodes in the network and potential mediators of apigenin-G’s effects on AS. KEGG pathway analysis of the intersecting targets revealed the “Lipid and atherosclerosis” pathway among the top 20 enriched pathways (Figure 7D), suggesting a potential mechanism for apigenin-G’s anti-atherosclerotic effects. To validate the involvement of the “Lipid and atherosclerosis” pathway, we examined the effect of apigenin-G on lipid levels in AS zebrafish. Results showed that apigenin-G significantly reduced lipid levels in AS zebrafish (Figure 7E), supporting the computational prediction of its involvement in lipid regulation. Among the top 10 targets, NF-κB1 and TP53 are involved in the “Lipid and atherosclerosis” pathway, and molecular docking studies have shown that the binding energy between NF-κB1 and TP53 and apigenin-G is less than 5 kcal/mol, indicating high affinity and stable binding (Figures 7F,G and Table 3; Supplementary Table S1). These in silico results suggest direct interactions between apigenin-G and key proteins in the atherosclerosis pathway.

**Table 3:** Binding energy between compounds and their targets.

Compound	Target	Binding energy (kcal/mol)
Apigenin-G	NF-κB	−9.7
	TP53	−9.8
Naringenin	BCL2	−7.3
	SRC	−6.3
	BCL2L1	−7.7
	GSK3β	−7.3
Dendrophenol	JUN	−4.9
	NFKB1	−6.3
	FOS	−5

Furthermore, qRT-PCR analysis demonstrated that the mRNA level of NF-κB1 was significantly upregulated in the AS group compared with controls and was significantly downregulated after apigenin-G treatment (Figure 7H, *p* < 0.001). Similarly, the expression of TP53 was decreased in AS zebrafish but significantly restored following apigenin-G administration (Figure 7I, *p* < 0.001). These gene expression changes provide molecular evidence supporting the regulatory effect of apigenin-G on NF-κB1 and TP53, key players in the “Lipid and atherosclerosis” pathway. Collectively, these findings support the potential role of apigenin-G in mitigating AS through modulation of this pathway.

### 3.3 Naringenin may alleviate AS through the “Lipid and atherosclerosis” pathway

**Figure 8:**
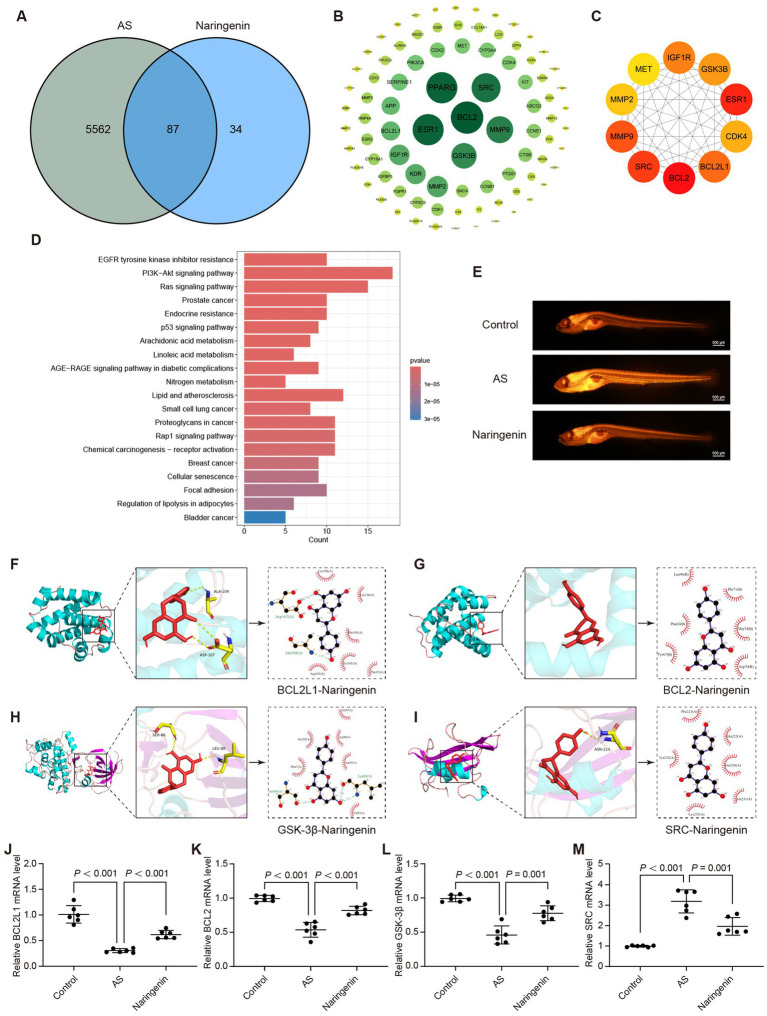
Naringenin may alleviate AS through its lipid-lowering effect. **(A)** Venn diagram showing the intersection between naringenin-related targets and AS-related targets, identifying 87 common targets potentially involved in its therapeutic effect. **(B)** PPI network of naringenin with AS-related targets, where green nodes represent significant proteins, and the size of the nodes indicates their importance. **(C)** The top 10 core targets identified by Cytoscape, including BCL2, GSK3B, and SRC, which play a crucial role in the lipid metabolism pathways associated with AS. **(D)** KEGG pathway analysis showing the top 20 pathways associated with naringenin, with significant pathways like “Lipid and atherosclerosis,” indicating its role in lipid modulation. **(E)** Nile Red staining results showing that naringenin significantly reduced lipid accumulation in the blood vessels of AS zebrafish, demonstrating its lipid-lowering properties. Scale bar = 500 μm. **(F)** Molecular docking of naringenin with BCL2L1, showing strong binding interaction, which may contribute to its anti-atherosclerotic effect. Similar molecular docking analyses show naringenin’s interactions with BCL2 **(G)**, GSK3β **(H)**, and SRC **(I)**. **(J–M)** qRT-PCR analysis of mRNA expression levels of core genes in zebrafish: **(J)** BCL2L1, **(K)** BCL2, **(L)** GSK-3β, and **(M)** SRC. Data are expressed as mean ± SD (*n* = 6).

Network pharmacology analysis was conducted to investigate the potential mechanism of naringenin in treating atherosclerosis (AS). The results revealed 87 overlapping targets between naringenin and AS (Figure 8A). A PPI network was constructed using the STRING database (Figure 8B). Cytoscape software was employed to identify the top 10 core targets based on the Maximal Clique Centrality (MCC) algorithm, including BCL2, ESR1, SRC, MMP9, BCL2L1, IGF1R, GSK3B, CDK4, MMP2, and MET (Figure 8C). KEGG pathway analysis of the intersecting targets yielded the top 20 pathways, with the “Lipid and atherosclerosis” pathway being particularly noteworthy (Figure 8D).

To elucidate whether naringenin mitigates AS through the “Lipid and atherosclerosis” pathway, we examined its effect on lipid levels in AS zebrafish. The results demonstrated that naringenin treatment significantly reduced lipid accumulation in AS zebrafish (Figure 8E), supporting its potential lipid-lowering effects *in vivo*. Among the top 10 targets, BCL2, SRC, BCL2L1, and GSK3β were found to be involved in the “Lipid and atherosclerosis” pathway. Molecular docking studies revealed (Figures 8F–I and Table 3; Supplementary Table S2).

To further investigate the regulatory role of naringenin on key genes within the “Lipid and atherosclerosis” pathway, we performed qRT-PCR to assess the expression levels of BCL2L1, BCL2, GSK3β, and SRC. Compared with the control group, all four genes were markedly downregulated in the AS model. Notably, naringenin administration significantly reversed these alterations, restoring gene expression to near-normal levels (Figures 8J–M, *p* < 0.001 or *p* = 0.001). These findings suggest that naringenin may mitigate AS progression by modulating the transcriptional activity of multiple targets implicated in lipid metabolism and vascular homeostasis.

### 3.4 Dendrophenol may alleviate AS through the “Lipid and atherosclerosis” pathway

**Figure 9:**
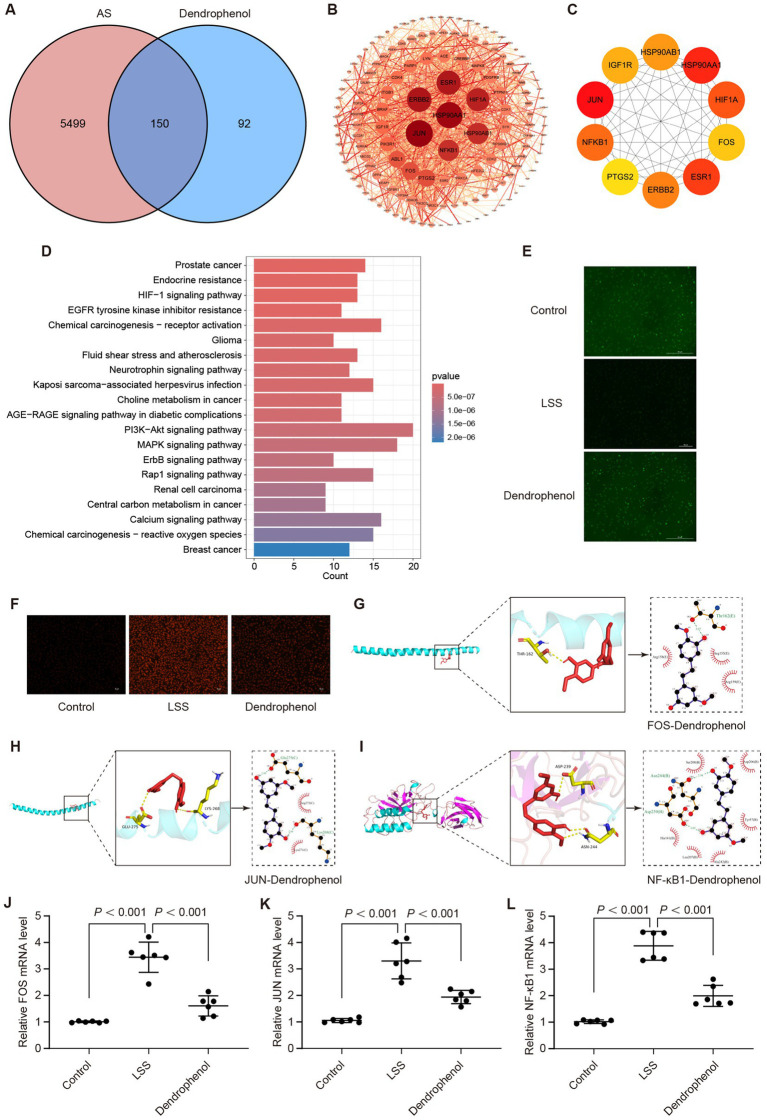
Dendrophenol may alleviate AS by improving LSS induced endothelial cell dysfunction. **(A)** Venn analysis between dendrophenol related targets and AS related targets, where the intersection represents the effective targets of dendrophenol after AS treatment. **(B)** PPI network. In the network, dark red nodes can be considered important, while light red nodes are less important, and the width of the edges indicates the strength of protein connections. **(C)** The top 10 important targets. **(D)** Top 20 clustering bar charts of KEGG pathway. **(E)** Dendrophenol increases the activity of NO in vascular endothelial cells (*n* = 3), scale bar: 100 μm. **(F)** Dendrophenol reduces the activity of ROS in vascular endothelial cells (*n* = 3), scale bar: 25 μm. **(G)** Molecular docking diagram of dendrophenol and FOS. **(H)** Molecular docking diagram of dendrophenol and JUN. **(I)** Molecular docking diagram of dendrophenol and NF-κB1. **(J–L)** qRT-PCR results showing mRNA expression levels of: **(J)** FOS, **(K)** JUN, and **(L)** NF-κB1. Data are presented as mean ± SD (*n* = 6).

Network pharmacology analysis was also employed to elucidate the potential mechanism of dendrophenol in treating AS. The results identified 150 overlapping targets between dendrophenol and AS (Figure 9A). The STRING database analyzed the intersecting targets and plotted a PPI graph (Figure 9B). Cytoscape analysis using the MCC algorithm revealed the top 10 core targets: JUN, HSP90AA1, ESR1, HIF1A, NF-κB1, ERBB2, HSP90AB1, IGF1R, FOS, and PTGS2 (Figure 9C). Perform KEGG analysis on the intersection targets to obtain the top 20 pathways, including “Fluid shear stress and atherosclerosis” pathway (Figure 9D).

To investigate whether dendrophenol alleviates AS through the “Fluid shear stress and atherosclerosis” pathway, we examined its effect on LSS-induced endothelial cell dysfunction. The results demonstrated that dendrophenol treatment significantly increased NO activity, which was decreased by LSS, and significantly reduced ROS activity, which was increased by LSS (Figures 9E,F). Among the top 10 targets, JUN, NF-κB1, and FOS are involved in the “Fluid shear stress and atherosclerosis” pathway, and molecular docking studies have shown that the binding energy between targets and dendrophenol is less than 5 kcal/mol, indicating high affinity and stable binding (Figures 9G–I and Table 3; Supplementary Table S3).

To further confirm these findings, we examined the transcriptional expression of FOS, JUN, and NF-κB1 using qRT-PCR. The results revealed that all three genes were significantly upregulated in the LSS model, but markedly downregulated upon dendrophenol treatment (Figures 9J–L, *p* < 0.001). These gene expression trends support the hypothesis that dendrophenol may attenuate endothelial dysfunction and inflammatory responses in AS through modulation of the “Fluid shear stress and atherosclerosis” pathway.

## 4 Discussion

AS remains a leading cause of cardiovascular diseases worldwide, with its multifactorial pathogenesis involving lipid accumulation, endothelial dysfunction, and inflammatory responses. While conventional treatments such as lipid-lowering drugs offer some relief, they often fail to provide a complete solution for AS management (15). This study explores a promising alternative for preventing and treating AS by evaluating the therapeutic effects of DH water extract and its active compounds, providing new insights into potential natural interventions for cardiovascular health.

The identification of six active compounds in DH water extract, namely, vicenin-1, schaftoside, apigenin-G, isoschaftoside, naringenin, and dendrophenol, provided a strong basis for investigating their individual contributions to AS therapy. Among these, apigenin-G, naringenin, and dendrophenol were selected for further study due to their promising effects in reducing cholesterol accumulation and modulating key cellular processes implicated in AS development.

Hyperlipidemia is widely recognized as a principal risk factor for AS (16, 17). Elevated lipid levels lead to plaque formation in blood vessels, contributing to vascular stenosis and impairing circulation (17). In our study, both apigenin-G and naringenin demonstrated significant lipid-lowering effects in zebrafish models of AS. Network pharmacology analyses revealed that these compounds may exert their therapeutic effects via the “Lipid and AS” pathway, which is known to regulate lipid metabolism and cholesterol homeostasis. Our experimental results, showing reduced lipid accumulation in zebrafish treated with these compounds, corroborate these *in silico* predictions. These findings align with previous studies highlighting the lipid-lowering properties of flavonoids such as apigenin and naringenin, which have been shown to regulate lipid metabolism in both animal models and clinical trials (18, 19).

On the other hand, the role of LSS in atherosclerosis pathogenesis is becoming increasingly evident. LSS, often present in regions of vascular curvature and bifurcations, promotes endothelial cell dysfunction by reducing NO production and increasing ROS generation (20). These changes contribute to endothelial damage and accelerate atherosclerotic plaque formation. Our study highlights the potential of dendrophenol, a bioactive compound in DH, in mitigating LSS-induced endothelial dysfunction. By increasing NO activity and reducing ROS production, dendrophenol appears to restore endothelial function and inhibit the progression of AS. The network pharmacology results further suggest that dendrophenol may act through the “Fluid shear stress and AS” pathway, influencing key mechanosensitive targets such as JUN, NF-κB1, and FOS (21–23). These findings are consistent with the growing body of literature emphasizing the importance of endothelial cell mechanotransduction in AS and the potential therapeutic benefits of compounds targeting shear stress-related pathways.

The zebrafish model used in this study offers a unique advantage for investigating the early stages of AS. The transparency of zebrafish embryos and larvae facilitates real-time visualization of vascular changes and lipid accumulation within blood vessels, making it an ideal model for studying the effects of therapeutic compounds. The rapid development and short lifespan of zebrafish also enable high-throughput screening of multiple compounds, as demonstrated by our evaluation of apigenin-G, naringenin, and dendrophenol.

However, it is important to acknowledge the limitations of using zebrafish as a model for AS research. While zebrafish are invaluable for studying the early stages of atherosclerosis, their vasculature lacks some of the complexities of human blood vessels, particularly in advanced stages of the disease (24). Moreover, zebrafish do not possess a fully developed immune system, which may affect the generalizability of results related to inflammation and immune responses in atherosclerosis. Nonetheless, zebrafish remain an excellent preclinical model for exploring the therapeutic potential of compounds and generating hypotheses for further validation in more complex mammalian systems.

Several additional limitations of this study must be considered. Firstly, the use of zebrafish models, while useful for early-stage AS research, limits the translation of our findings to human clinical outcomes due to the differences in vascular complexity and immune system development. Secondly, the small sample size may affect the statistical power of the results, necessitating larger-scale studies to confirm these findings. Thirdly, the absence of a dose–response evaluation precludes the determination of optimal therapeutic doses for DH extract and its active compounds. Finally, comprehensive toxicity assessments were not conducted, leaving the long-term safety profile of DH extract and its compounds unclear.

In conclusion, our study demonstrates the potential of *Dendrobium huoshanense* water extract and its active compounds, apigenin-G, naringenin, and dendrophenol, as effective candidates for preventing and treating atherosclerosis. These compounds exhibit lipid-lowering effects and improve endothelial function, thereby targeting key mechanisms involved in AS development. While our findings are encouraging, further research, particularly long-term studies and clinical trials in humans, is necessary to fully validate the efficacy and safety of these compounds. Nevertheless, the promising results of this study provide a strong foundation for the development of DH-based functional foods or nutraceuticals for AS prevention and treatment.

## 5 Conclusion

This study provides compelling evidence for the potential of *Dendrobium huoshanense* water extract and its active compounds, apigenin-G, naringenin, and dendrophenol, as promising candidates for the prevention and treatment of AS. Using the zebrafish model, we demonstrated that these compounds significantly reduce lipid accumulation and improve endothelial dysfunction, both key factors in the development of AS. Apigenin-G and naringenin appear to exert therapeutic effects by modulating lipid metabolism through the “Lipid and atherosclerosis” pathway, while dendrophenol shows promise in improving endothelial dysfunction induced by low shear stress via the “Fluid shear stress and atherosclerosis” pathway.

Our findings highlight the multifaceted mechanisms through which DH and its active constituents may contribute to the prevention and management of AS, supporting their potential role as functional foods or nutraceuticals. The use of multiple active compounds targeting different aspects of atherosclerosis pathogenesis represents a novel approach that may offer synergistic benefits in disease prevention and treatment. However, these results are based on preclinical models, and further studies, including long-term clinical trials and investigations into the complete range of bioactive components within DH, are necessary to fully establish its efficacy and safety in human populations. While DH holds promise as a natural intervention for cardiovascular health, its clinical application will require further validation.

## Data Availability

The original contributions presented in the study are included in the article/Supplementary material, further inquiries can be directed to the corresponding authors.

## Footnotes

^1^ https://www.genecards.org/

^2^ https://www.omim.org/

^3^ https://www.disgenet.org/

^4^ http://swisstargetprediction.ch/

^5^ https://sea.bkslab.org/

^6^ https://metascape.org/gp/index.html

^7^ http://www.Bioinformatics.com.cn/srplot

^8^ https://www.rcsb.org/
