# Supplementary material for: Dendrobium huoshanense C. Z. Tang & S. J. Cheng alleviates atherosclerosis by reducing lipid and improving vascular endothelial dysfunction
Source: Front Nutr. 2025 Sep 26;12:1649161. doi: 10.3389/fnut.2025.1649161 (PMC12510864; doi:10.3389/fnut.2025.1649161)
Supplement: Supplementary file 1 [file Data_Sheet_1.pdf]

Supplementary Materials

Original Article

Dendrobium huoshanense C. Z. Tang & S. J. Cheng alleviates atherosclerosis by reducing lipid and improving vascular endothelial dysfunction

Xiang-Cheng Fan[1,7#], Wei-You Cao[2,#], Minyang He[5,#], Hui-Kai Wang[4], Qing Hao[2], Wen-Jing Liu[2], Zhao-Ying Ren[2], Li-Jun Wang[2], Jing-Yu Wang[2], Fei-Xue Wang[2], Lin Jiang[2], Qiu-Sheng Zheng[2], Jun Ma[2], Feng Zhang[6,*], Ji-Chun Han[2,*], Lei Zheng[3,*]

[1]*Department of Pharmacy, Center for Membrane Receptor and Brain Medicine, The Fourth Affiliated Hospital of School of Medicine, and International School of Medicine, International Institutes of Medicine, Zhejiang University, Yiwu, China, 322000*

[2]*College of Traditional Chinese Medicine, Binzhou Medical University, Yantai, China, 264003*

[3]*Department of Cardiovascular Surgery, Yantai Yuhuangding Hospital, Yantai, China, 264099*

[4]*Binzhou Medical University Affiliated Traditional Chinese Medicine Hospital, Binzhou Medical University, Binzhou, China, 256601*

[5]*Department of Gastroenterology, The Fourth Affiliated Hospital of School of Medicine, and International School of Medicine, International Institutes of Medicine, Zhejiang University, Yiwu,China,322000*

[6]*Department of Pharmacy, Changzheng Hospital, Naval Medical University, Shanghai, China, 200003*

[7]*Center for Innovative Traditional Chinese Medicine Target and New Drug Research, International Institutes of Medicine, Zhejiang University, Yiwu, China, 322000*

[#]These authors contributed equally.

Address for correspondence: ytzhenglei@sina.com (L.Z), 923023681@qq.com (J.H) and fengzhang@smmu.edu.cn (F.Z)

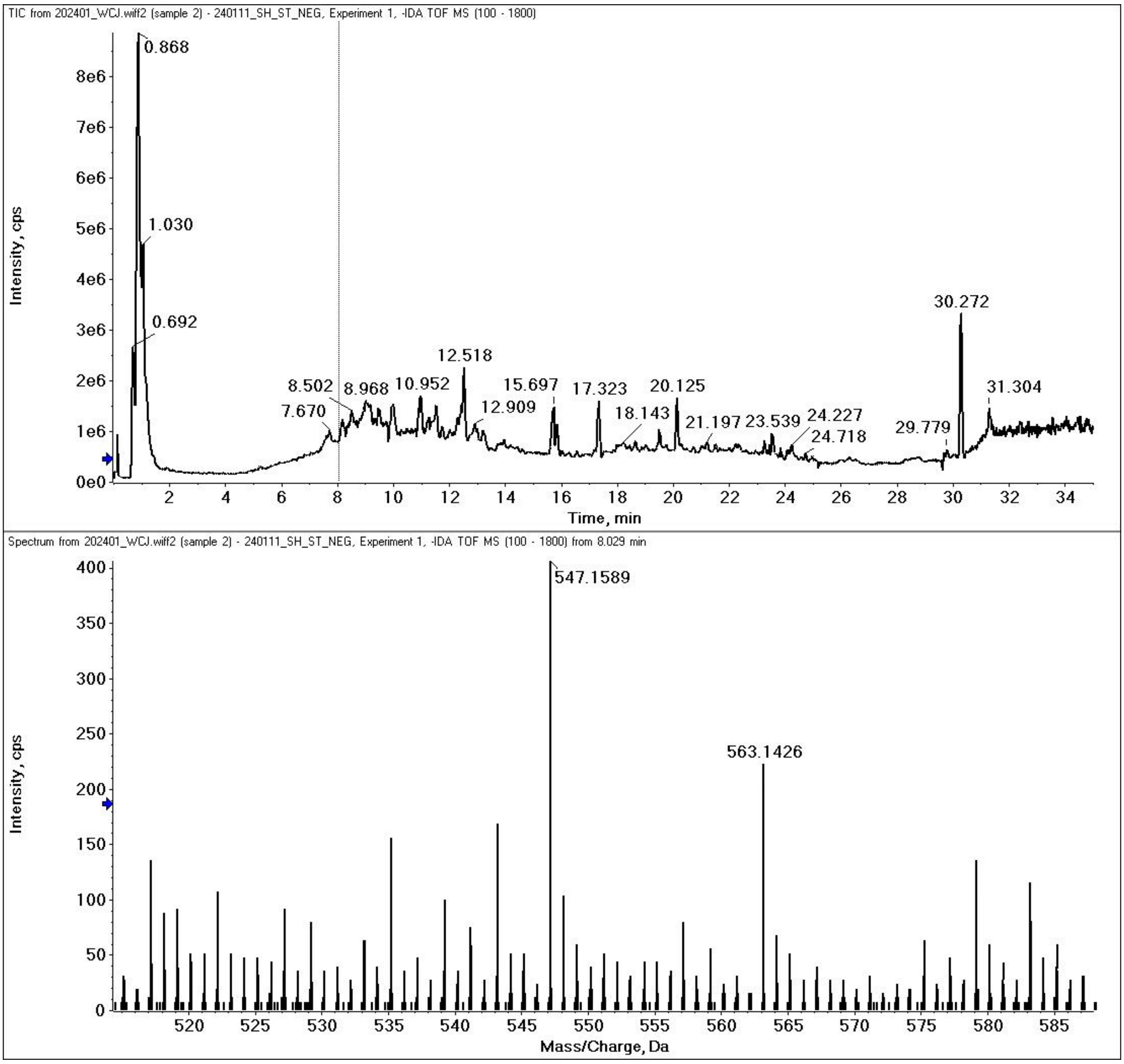

Fig S1. Primary mass spectrum of DH water extract 1 (Vicenin-1).

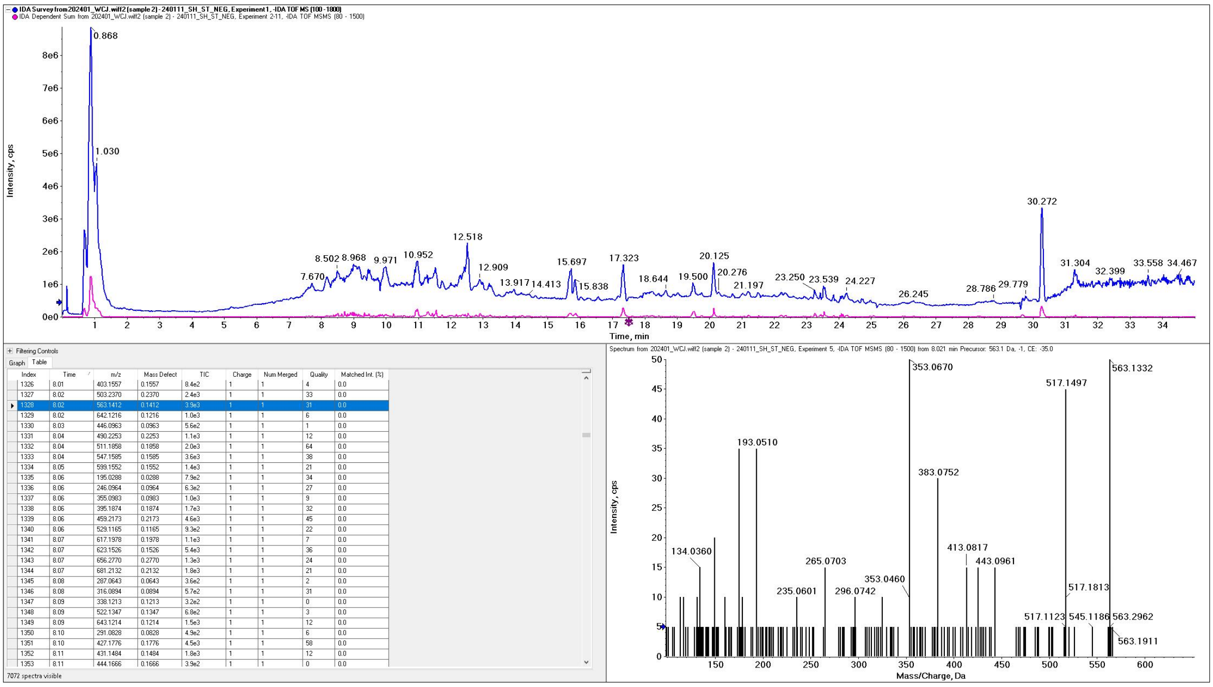

Index	Time	m/z	Mass Defect	TIC	Charge	Num Merged	Quality	Matched Int. (%)
1326	8.01	403.1557	0.1557	8.4e2	1	1	4	0.0
1327	8.02	503.2370	0.2370	2.4e3	1	1	33	0.0
1328	8.02	563.1412	0.1412	3.9e3	1	1	31	0.0
1329	8.02	642.1216	0.1216	1.0e3	1	1	6	0.0
1330	8.03	446.0963	0.0963	5.6e2	1	1	1	0.0
1331	8.04	490.2253	0.2253	1.1e3	1	1	12	0.0
1332	8.04	511.1858	0.1858	2.0e3	1	1	64	0.0
1333	8.04	547.1585	0.1585	3.6e3	1	1	38	0.0
1334	8.05	599.1552	0.1552	1.4e3	1	1	21	0.0
1335	8.06	195.0288	0.0288	7.9e2	1	1	34	0.0
1336	8.06	246.0964	0.0964	6.3e2	1	1	27	0.0
1337	8.06	355.0983	0.0983	1.0e3	1	1	9	0.0
1338	8.06	395.1874	0.1874	1.7e3	1	1	32	0.0
1339	8.06	459.2173	0.2173	4.6e3	1	1	45	0.0
1340	8.06	529.1165	0.1165	9.3e2	1	1	22	0.0
1341	8.07	617.1978	0.1978	1.1e3	1	1	7	0.0
1342	8.07	623.1526	0.1526	5.4e3	1	1	36	0.0
1343	8.07	656.2770	0.2770	1.3e3	1	1	24	0.0
1344	8.07	681.2132	0.2132	1.8e3	1	1	21	0.0
1345	8.08	287.0643	0.0643	3.6e2	1	1	2	0.0
1346	8.08	316.0894	0.0894	5.7e2	1	1	31	0.0
1347	8.09	338.1213	0.1213	3.2e2	1	1	0	0.0
1348	8.09	522.1347	0.1347	6.8e2	1	1	3	0.0
1349	8.09	643.1214	0.1214	1.5e3	1	1	12	0.0
1350	8.10	291.0828	0.0828	4.9e2	1	1	6	0.0
1351	8.10	427.1776	0.1776	4.5e3	1	1	58	0.0
1352	8.11	431.1484	0.1484	1.8e3	1	1	12	0.0
1353	8.11	444.1666	0.1666	3.9e2	1	1	0	0.0

Fig S2. Secondary Mass Spectrometry of DH Water Extract 1 (Vicenin-1).

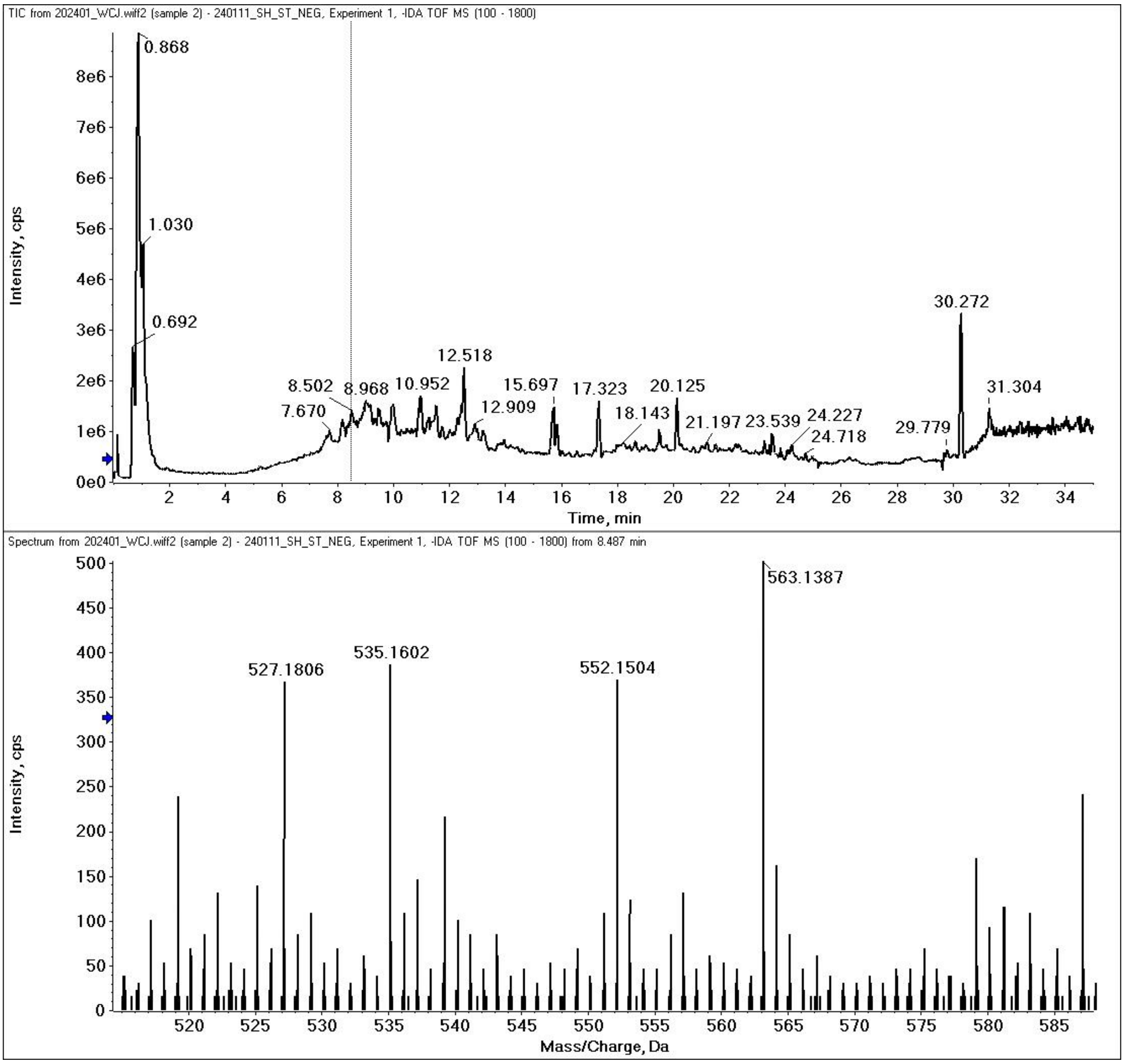

Fig S3. Primary mass spectrum of DH water extract 2 (Schaftoside).

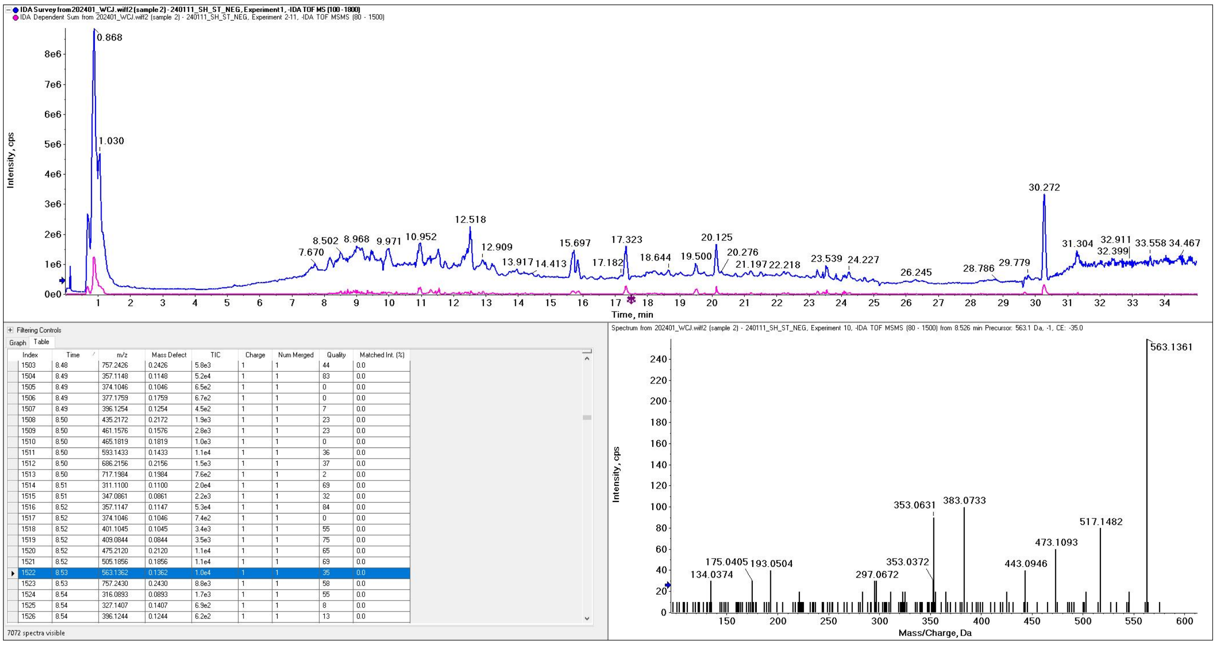

Index	Time	m/z	Mass Defect	TIC	Charge	Num Merged	Quality	Matched Int. (%)
1503	8.48	757.2426	0.2426	5.8e3	1	1	44	0.0
1504	8.49	357.1148	0.1148	5.2e4	1	1	83	0.0
1505	8.49	374.1046	0.1046	6.5e2	1	1	0	0.0
1506	8.49	377.1759	0.1759	6.7e2	1	1	0	0.0
1507	8.49	396.1254	0.1254	4.5e2	1	1	7	0.0
1508	8.50	435.2172	0.2172	1.9e3	1	1	23	0.0
1509	8.50	461.1576	0.1576	2.8e3	1	1	23	0.0
1510	8.50	465.1819	0.1819	1.0e3	1	1	0	0.0
1511	8.50	593.1433	0.1433	1.1e4	1	1	36	0.0
1512	8.50	686.2156	0.2156	1.5e3	1	1	37	0.0
1513	8.50	717.1984	0.1984	7.6e2	1	1	2	0.0
1514	8.51	311.1100	0.1100	2.0e4	1	1	69	0.0
1515	8.51	347.0861	0.0861	2.2e3	1	1	32	0.0
1516	8.52	357.1147	0.1147	5.3e4	1	1	84	0.0
1517	8.52	374.1046	0.1046	7.4e2	1	1	0	0.0
1518	8.52	401.1045	0.1045	3.4e3	1	1	55	0.0
1519	8.52	409.0844	0.0844	3.5e3	1	1	75	0.0
1520	8.52	475.2120	0.2120	1.1e4	1	1	65	0.0
1521	8.52	505.1856	0.1856	1.1e4	1	1	69	0.0
1522	8.53	563.1362	0.1362	1.0e4	1	1	35	0.0
1523	8.53	757.2430	0.2430	8.8e3	1	1	58	0.0
1524	8.54	316.0893	0.0893	1.7e3	1	1	55	0.0
1525	8.54	327.1407	0.1407	6.9e2	1	1	8	0.0
1526	8.54	396.1244	0.1244	6.2e2	1	1	13	0.0

Fig S4. Secondary Mass Spectrometry of DH Water Extract 2 (Schaftoside).

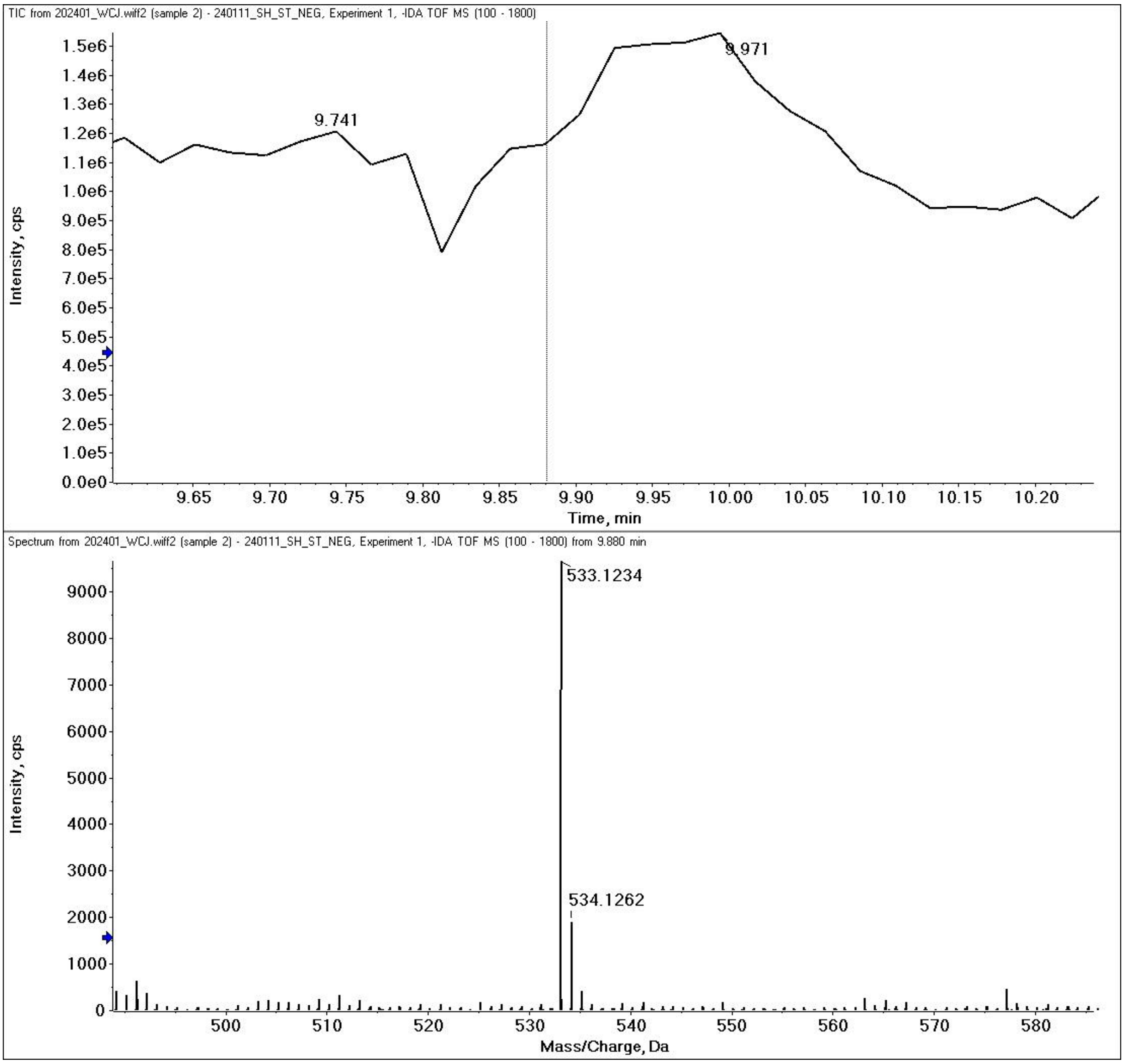

Fig S5. Primary mass spectrum of DH water extract 3 (Apigenin-G).

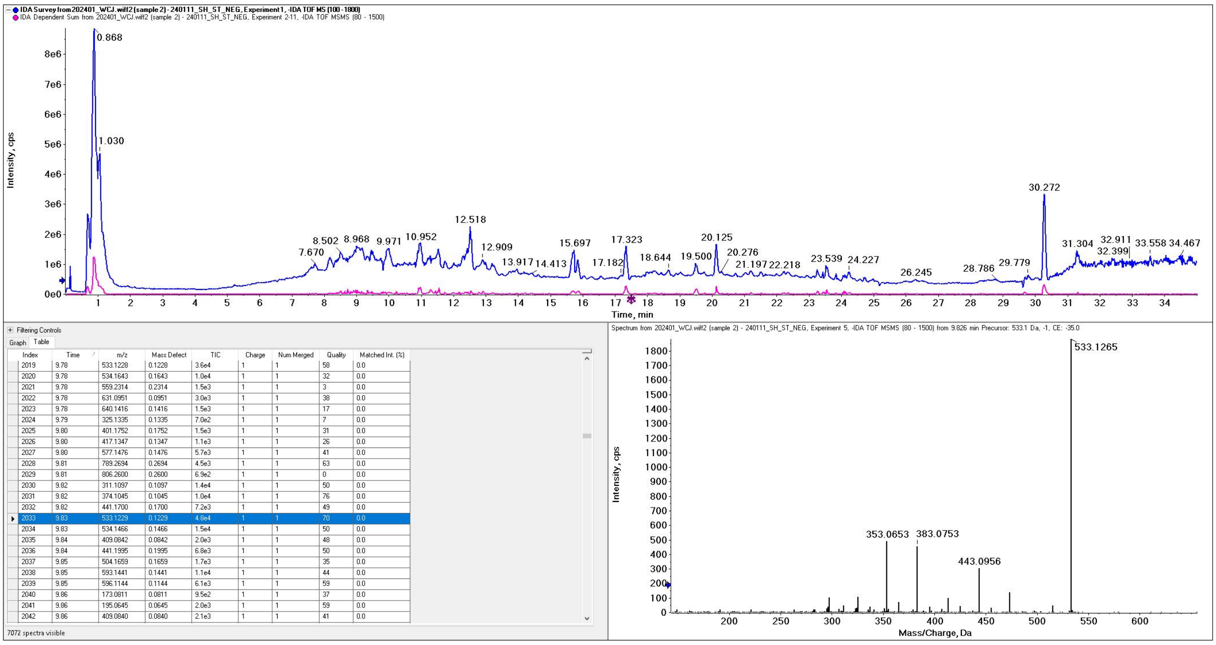

Index	Time	m/z	Mass Defect	TIC	Charge	Num Merged	Quality	Matched Int. (%)
2019	9.78	533.1228	0.1228	3.6e4	1	1	58	0.0
2020	9.78	534.1643	0.1643	1.0e4	1	1	32	0.0
2021	9.78	559.2314	0.2314	1.5e3	1	1	3	0.0
2022	9.78	631.0951	0.0951	3.0e3	1	1	38	0.0
2023	9.78	640.1416	0.1416	1.5e3	1	1	17	0.0
2024	9.79	325.1335	0.1335	7.0e2	1	1	7	0.0
2025	9.80	401.1752	0.1752	1.5e3	1	1	31	0.0
2026	9.80	417.1347	0.1347	1.1e3	1	1	26	0.0
2027	9.80	577.1476	0.1476	5.7e3	1	1	41	0.0
2028	9.81	789.2694	0.2694	4.5e3	1	1	63	0.0
2029	9.81	806.2600	0.2600	6.9e2	1	1	0	0.0
2030	9.82	311.1097	0.1097	1.4e4	1	1	50	0.0
2031	9.82	374.1045	0.1045	1.0e4	1	1	76	0.0
2032	9.82	441.1700	0.1700	7.2e3	1	1	49	0.0
2033	9.83	533.1229	0.1229	4.8e4	1	1	70	0.0
2034	9.83	534.1466	0.1466	1.5e4	1	1	50	0.0
2035	9.84	409.0842	0.0842	2.0e3	1	1	48	0.0
2036	9.84	441.1995	0.1995	6.8e3	1	1	50	0.0
2037	9.85	504.1659	0.1659	1.7e3	1	1	35	0.0
2038	9.85	593.1441	0.1441	1.1e4	1	1	44	0.0
2039	9.85	596.1144	0.1144	6.1e3	1	1	59	0.0
2040	9.86	173.0811	0.0811	9.5e2	1	1	37	0.0
2041	9.86	195.0645	0.0645	2.0e3	1	1	59	0.0
2042	9.86	409.0840	0.0840	2.1e3	1	1	41	0.0

Fig S6. Secondary Mass Spectrometry of DH Water Extract 3 (Apigenin-G).

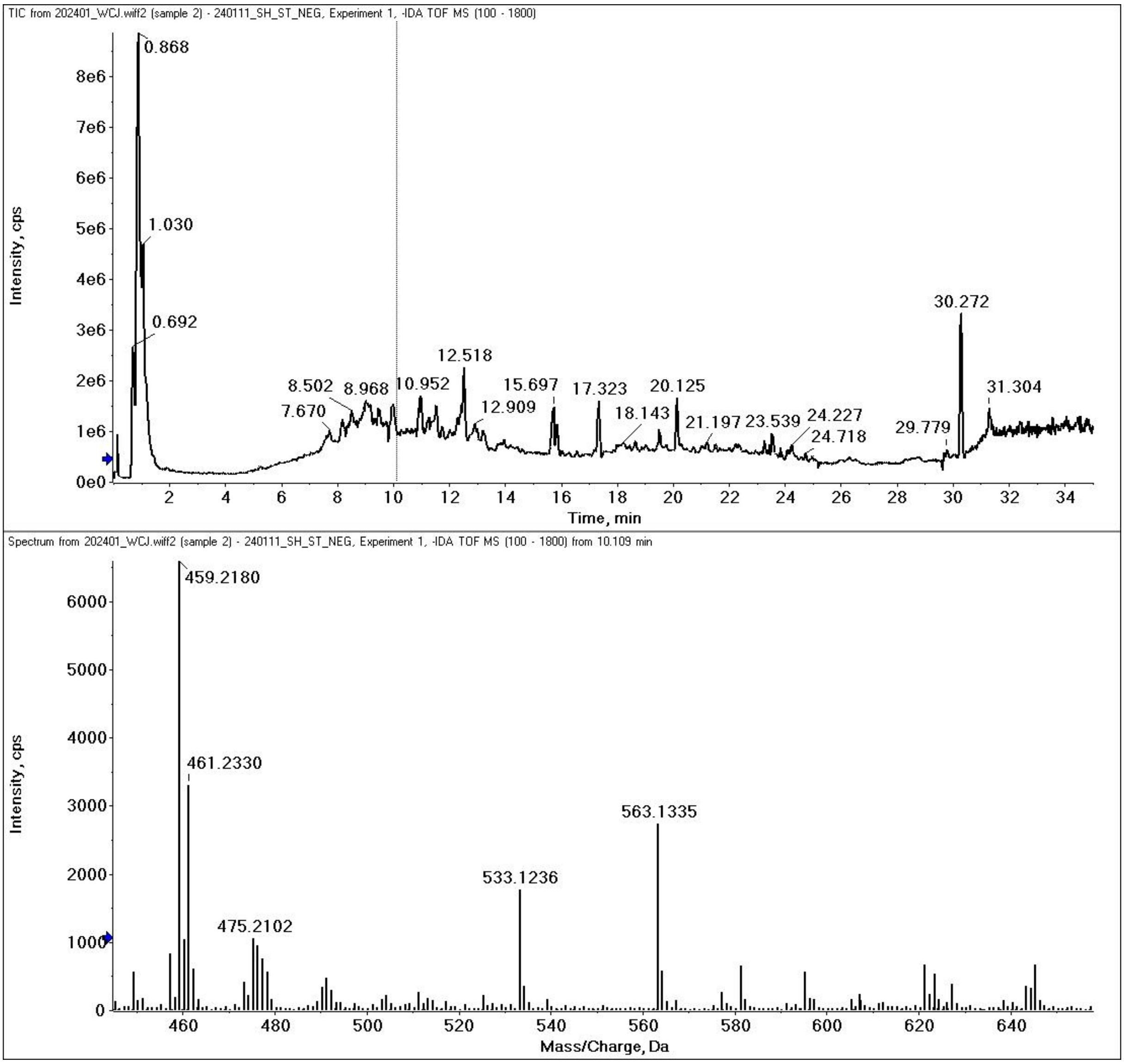

Fig S7. Primary mass spectrum of DH water extract 4 (Isoschaftoside).

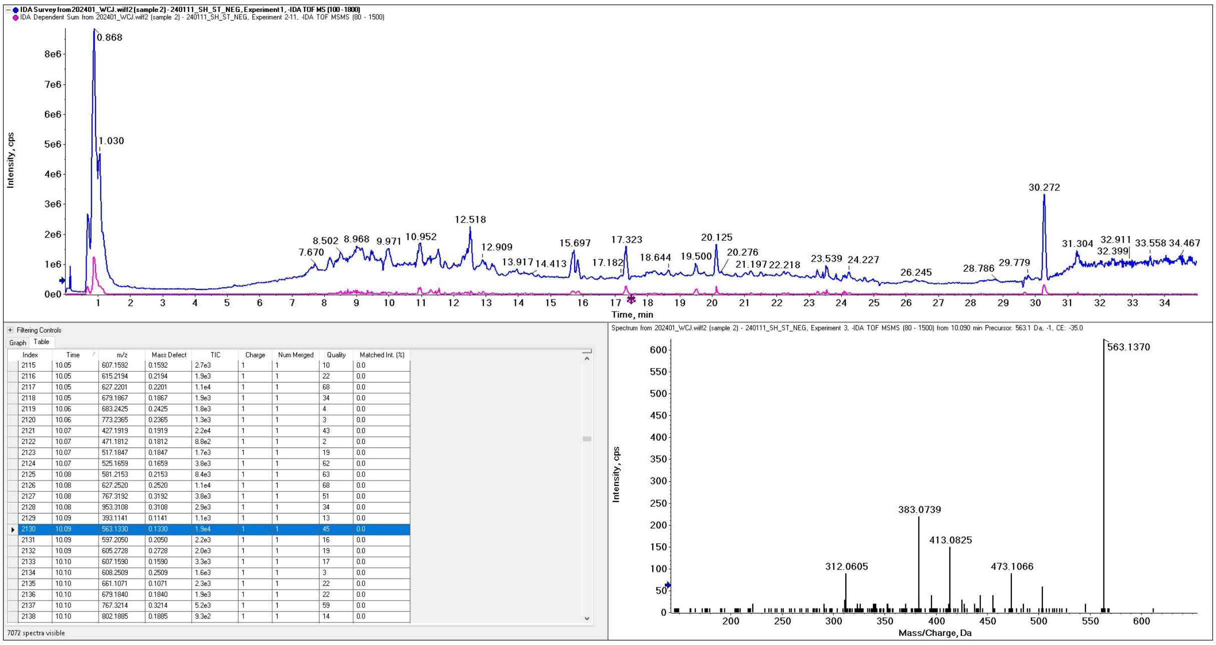

Index	Time	m/z	Mass Defect	TIC	Charge	Num Merged	Quality	Matched Int. (%)
2115	10.05	607.1592	0.1592	2.7e3	1	1	10	0.0
2116	10.05	615.2194	0.2194	1.9e3	1	1	22	0.0
2117	10.05	627.2201	0.2201	1.1e4	1	1	68	0.0
2118	10.05	679.1867	0.1867	1.9e3	1	1	34	0.0
2119	10.06	683.2425	0.2425	1.8e3	1	1	4	0.0
2120	10.06	773.2365	0.2365	1.3e3	1	1	3	0.0
2121	10.07	427.1919	0.1919	2.2e4	1	1	43	0.0
2122	10.07	471.1812	0.1812	8.8e2	1	1	2	0.0
2123	10.07	517.1847	0.1847	1.7e3	1	1	19	0.0
2124	10.07	525.1659	0.1659	3.8e3	1	1	62	0.0
2125	10.08	581.2153	0.2153	8.4e3	1	1	63	0.0
2126	10.08	627.2520	0.2520	1.1e4	1	1	68	0.0
2127	10.08	767.3192	0.3192	3.8e3	1	1	51	0.0
2128	10.08	953.3108	0.3108	2.9e3	1	1	34	0.0
2129	10.09	393.1141	0.1141	1.1e3	1	1	13	0.0
2130	10.09	563.1330	0.1330	1.9e4	1	1	45	0.0
2131	10.09	597.2050	0.2050	2.2e3	1	1	16	0.0
2132	10.09	605.2728	0.2728	2.0e3	1	1	19	0.0
2133	10.10	607.1590	0.1590	3.3e3	1	1	17	0.0
2134	10.10	608.2509	0.2509	1.6e3	1	1	3	0.0
2135	10.10	661.1071	0.1071	2.3e3	1	1	22	0.0
2136	10.10	679.1840	0.1840	1.9e3	1	1	22	0.0
2137	10.10	767.3214	0.3214	5.2e3	1	1	59	0.0
2138	10.10	802.1885	0.1885	9.3e2	1	1	14	0.0

Fig S8. Secondary Mass Spectrometry of DH Water Extract 4 (Isoschaftoside).

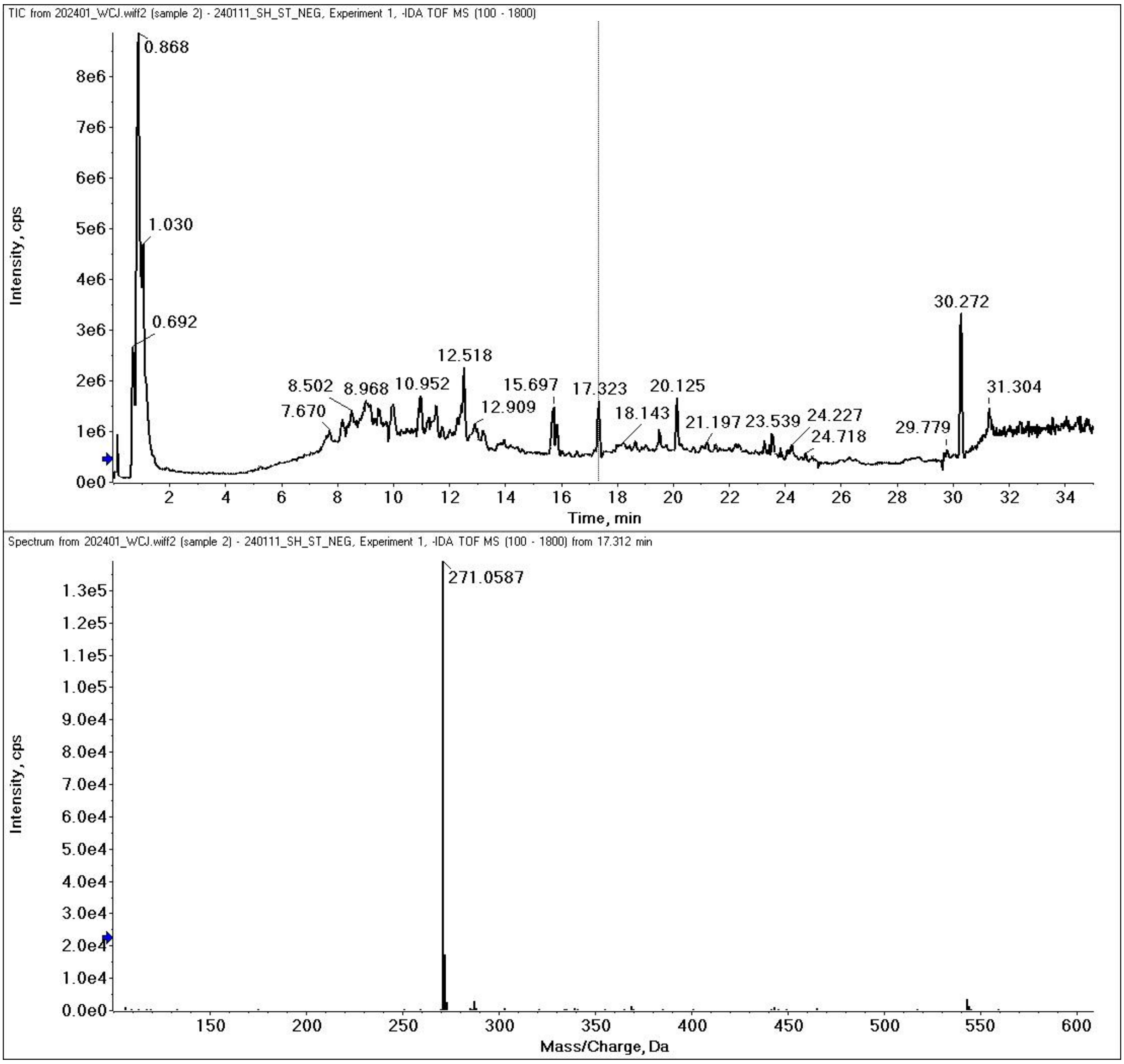

Fig S9. Primary mass spectrum of DH water extract 5 (Naringenin).

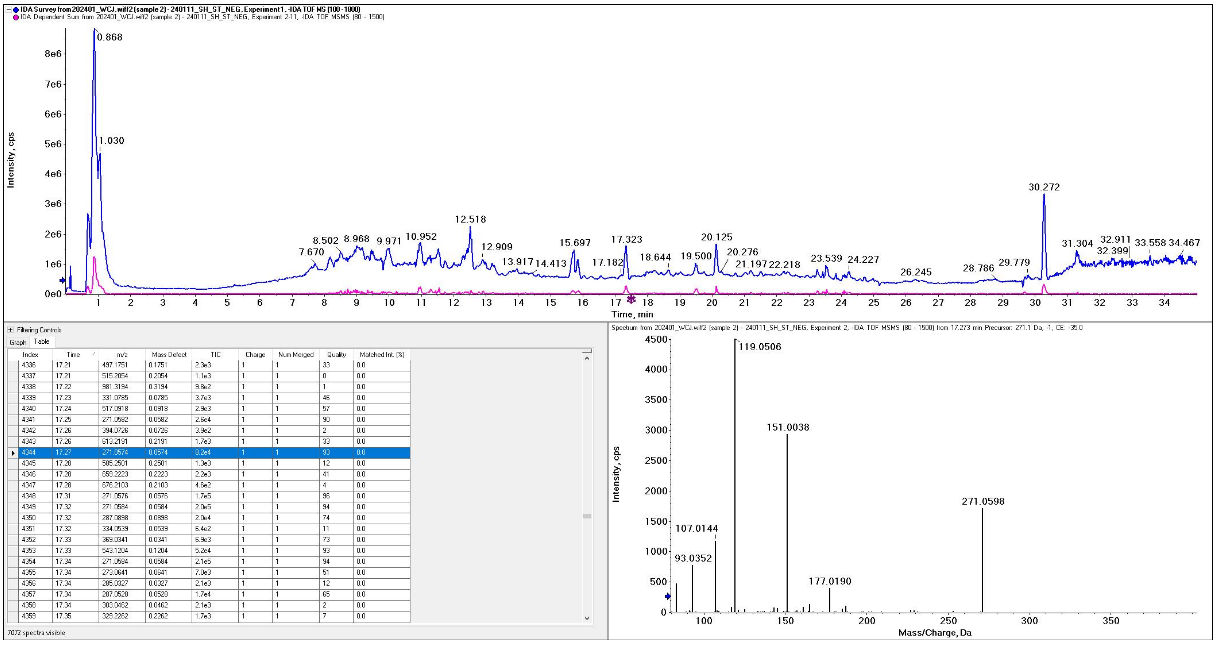

Index	Time	m/z	Mass Defect	TIC	Charge	Num Merged	Quality	Matched Int. (%)
4336	17.21	497.1751	0.1751	2.3e3	1	1	33	0.0
4337	17.21	515.2054	0.2054	1.1e3	1	1	0	0.0
4338	17.22	981.3194	0.3194	9.8e2	1	1	1	0.0
4339	17.23	331.0785	0.0785	3.7e3	1	1	46	0.0
4340	17.24	517.0918	0.0918	2.9e3	1	1	57	0.0
4341	17.25	271.0582	0.0582	2.6e4	1	1	90	0.0
4342	17.26	394.0726	0.0726	3.9e2	1	1	2	0.0
4343	17.26	613.2191	0.2191	1.7e3	1	1	33	0.0
4344	17.27	271.0574	0.0574	8.2e4	1	1	93	0.0
4345	17.28	585.2501	0.2501	1.3e3	1	1	12	0.0
4346	17.28	659.2223	0.2223	2.2e3	1	1	41	0.0
4347	17.28	676.2103	0.2103	4.6e2	1	1	4	0.0
4348	17.31	271.0576	0.0576	1.7e5	1	1	96	0.0
4349	17.32	271.0584	0.0584	2.0e5	1	1	94	0.0
4350	17.32	287.0898	0.0898	2.0e4	1	1	74	0.0
4351	17.32	334.0539	0.0539	6.4e2	1	1	11	0.0
4352	17.33	369.0341	0.0341	6.9e3	1	1	73	0.0
4353	17.33	543.1204	0.1204	5.2e4	1	1	93	0.0
4354	17.34	271.0584	0.0584	2.1e5	1	1	94	0.0
4355	17.34	273.0641	0.0641	7.0e3	1	1	51	0.0
4356	17.34	285.0327	0.0327	2.1e3	1	1	12	0.0
4357	17.34	287.0528	0.0528	1.7e4	1	1	65	0.0
4358	17.34	303.0462	0.0462	2.1e3	1	1	2	0.0
4359	17.35	329.2262	0.2262	1.7e3	1	1	7	0.0

Fig S10. Secondary Mass Spectrometry of DH Water Extract 5 (Naringenin).

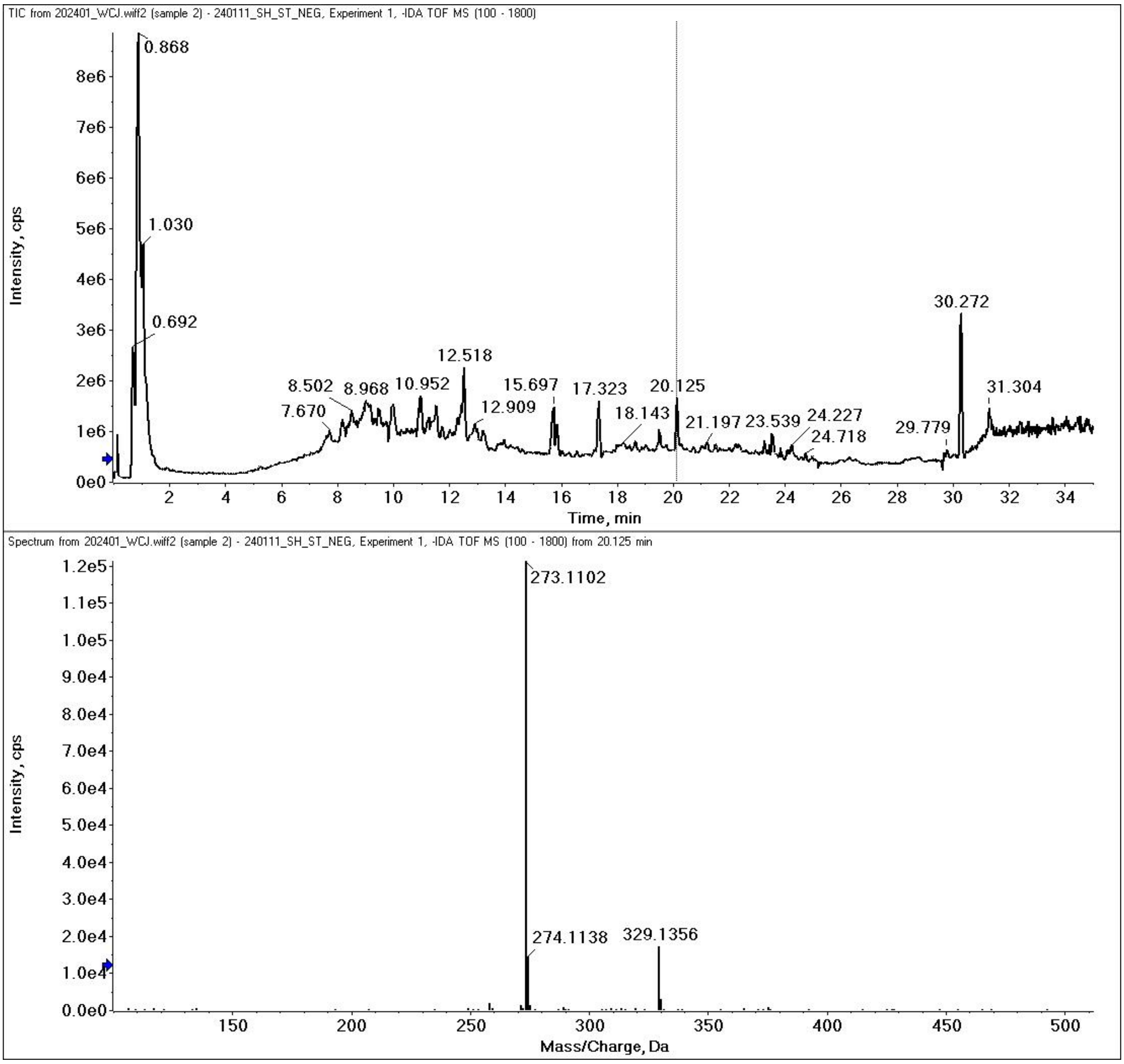

Fig S11. Primary mass spectrum of DH water extract 6 (Gigantol).

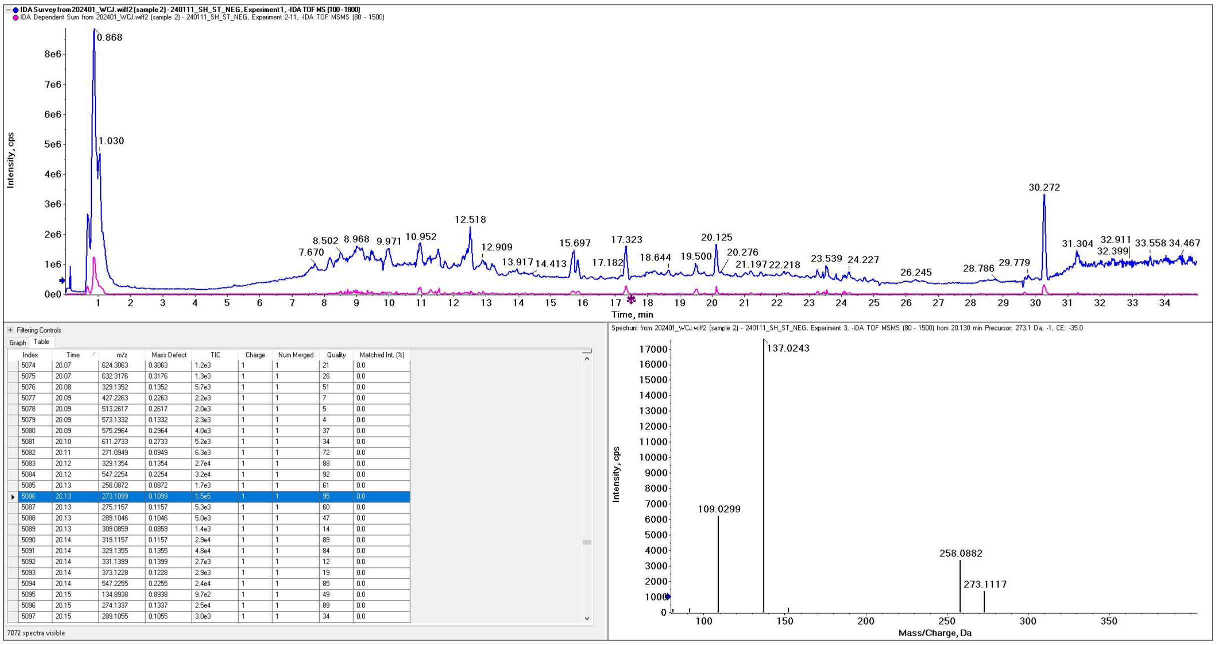

Fig S12. Secondary Mass Spectrometry of DH Water Extract 6 (Gigantol).

Table S1. Top 20 clustering bar charts of KEGG pathway (Apigenin-G).

Description	Gene ID
Hepatitis B	CREB1/CCNA2/TP53/PRKCG/PRKCB/MAP2K3/JAK3/MAPK8/MAPK10/MAP2K4/JAK2/CDK2/IRAK1/IKBKE/TYK2/IRAK4/CHUK/STAT1/TLR4/NFKB1/TBK1
Measles	IL2/TP53/JAK3/MAPK8/CDK6/GSK3B/MAPK10/CDK2/CDK4/IRAK1/IKBKE/TYK2/IRAK4/CSNK2B/CHUK/STAT1/TLR4/NFKB1/TBK1
Lipid and atherosclerosis	CALM1/TP53/MAP2K3/MAPK8/GSK3B/MAPK10/CAMK2D/MAP2K4/ROCK2/IRAK1/IKBKE/IRAK4/CAMK2G/CAMK2B/CHUK/TLR4/NFE2L2/NFKB1/TBK1
Yersinia infection	IL2/MAP2K3/MAPK8/RPS6KA1/GSK3B/MAPK10/MAP2K4/ROCK2/ROCK1/IRAK1/PKN1/IRAK4/CHUK/ZAP70/TLR4/ITGB1/NFKB1/TBK1
Glioma	CALM1/TP53/PRKCG/PRKCB/PDGFRA/EGFR/CAMK4/CDK6/CAMK2D/CDK4/CAMK2G/CAMK2B/PDGFRB/MTOR
Kaposi sarcoma-associated herpesvirus infection	CREB1/CALM1/VEGFA/TP53/MAPK8/CDK6/GSK3B/MAPK10/MAP2K4/JAK2/CDK4/IKBKE/TYK2/HIF1A/CHUK/MAPKAPK2/STAT1/MTOR/NFKB1/TBK1
PI3K-Akt signaling pathway	CREB1/IL2/VEGFA/TP53/CSF1R/KIT/PDGFRA/EGFR/EPHA2/JAK3/CDK6/GSK3B/JAK2/ERBB4/CDK2/CDK4/PKN1/PRKAA1/CHUK/TLR4/PDGFRB/HSP90AA1/MTOR/ITGB1/NFKB1/HSP90AB1
Human cytomegalovirus infection	CREB1/CALM1/VEGFA/TP53/PRKCG/PRKCB/PRKACA/PDGFRA/EGFR/CDK6/GSK3B/ROCK2/ROCK1/CDK4/CXCR4/CHUK/STING1/MTOR/PTGER1/NFKB1/TBK1
Neurotrophin signaling pathway	CALM1/TP53/ABL1/MAPK8/CAMK4/RPS6KA1/GSK3B/MAPK10/CAMK2D/IRAK1/IRAK4/CAMK2G/CAMK2B/MAPKAPK2/PTPN11/NFKB1
Alcoholic liver disease	MAP2K3/MAPK8/GSK3B/MAPK10/MAP2K4/IRAK1/IKBKE/IRAK4/PRKAA1/CAMKK2/CHUK/TLR4/ACACA/C5AR1/SCD/NFKB1/TBK1
MAPK signaling pathway	VEGFA/TP53/PRKCG/PRKCB/PRKACA/CSF1R/KIT/PDGFRA/EGFR/EPHA2/MAP2K3/MAPK8/RPS6KA1/MAPK10/MAP2K4/ERBB4/IRAK1/IRAK4/CHUK/MAPKAPK2/PDGFRB/NFKB1
ErbB signaling pathway	PRKCG/PRKCB/ABL1/EGFR/MAPK8/GSK3B/MAPK10/CAMK2D/MAP2K4/ERBB4/CAMK2G/CAMK2B/MTOR
Toll-like receptor signaling pathway	MAP2K3/MAPK8/MAPK10/MAP2K4/IRAK1/IKBKE/TYK2/IRAK4/CHUK/STAT1/TLR4/NFKB1/TLR8/TBK1
Th17 cell differentiation	IL2/JAK3/MAPK8/MAPK10/JAK2/TYK2/HIF1A/CHUK/ZAP70/STAT1/HSP90AA1/MTOR/NFKB1/HSP90AB1
Epstein-Barr virus infection	CCNA2/TP53/MAP2K3/JAK3/MAPK8/CDK6/MAPK10/MAP2K4/CDK2/CDK4/IRAK1/IKBKE/TYK2/IRAK4/CHUK/STAT1/NFKB1/TBK1
Ras signaling pathway	CALM1/VEGFA/PRKCG/PRKCB/PRKACA/CSF1R/ABL1/KIT/PDGFRA/EGFR/EPHA2/MAPK8/MAPK10/CHUK/ZAP70/PDGFRB/PTPN11/NFKB1/TBK1
Coronavirus disease - COVID-19	IL2/PRKCG/PRKCB/EGFR/MAPK8/MAPK10/IRAK1/IKBKE/TYK2/IRAK4/CHUK/F13A1/STAT1/STING1/TLR4/C5AR1/NFKB1/TLR8/TBK1
Proteoglycans in cancer	VEGFA/TP53/PRKCG/PRKCB/PRKACA/EGFR/CAMK2D/ROCK2/ERBB4/ROCK1/CAMK2G/CAMK2B/HIF1A/TLR4/MTOR/PTPN11/ITGB1
Insulin resistance	CREB1/PRKCB/PRKCZ/MAPK8/RPS6KA1/GSK3B/MAPK10/PYGM/PRKAA1/SLC2A1/MTOR/PTPN11/NFKB1
PD-L1 expression and PD-1 checkpoint pathway in cancer	EGFR/MAP2K3/JAK2/CSNK2B/HIF1A/CHUK/ZAP70/STAT1/TLR4/MTOR/PTPN11/NFKB1

Table S2. Top 20 clustering bar charts of KEGG pathway (Naringenin).

Description	Gene ID
EGFR tyrosine kinase inhibitor resistance	VEGFA/SRC/KDR/MET/IGF1R/BCL2L1/PIK3CB/PIK3CA/GSK3B/BCL2
PI3K-Akt signaling pathway	VEGFA/CDK2/RXRA/KIT/KDR/FGFR1/MET/IGF1R/INSR/BCL2L1/PIK3CB/PIK3CA/PGF/GSK3B/BCL2/SYK/CCNE1/CDK4
Ras signaling pathway	VEGFA/PLA2G1B/PLA2G2A/PLA2G5/PLA2G10/KIT/KDR/FGFR1/MET/IGF1R/INSR/BCL2L1/PIK3CB/PIK3CA/PGF
Prostate cancer	CDK2/FGFR1/IGF1R/PIK3CB/PIK3CA/MMP3/GSK3B/BCL2/MMP9/CCNE1
Endocrine resistance	ESR2/ESR1/SRC/IGF1R/MMP2/PIK3CB/PIK3CA/BCL2/MMP9/CDK4
p53 signaling pathway	CDK2/CCNB1/SERPINE1/IGFBP3/BCL2L1/BCL2/CCNE1/CDK4/CDK1
Arachidonic acid metabolism	CBR1/PTGS1/PLA2G1B/PLA2G2A/PLA2G5/PLA2G10/CYP2C9/ALOX12
Linoleic acid metabolism	PLA2G1B/PLA2G2A/PLA2G5/PLA2G10/CYP2C9/CYP3A4
AGE-RAGE signaling pathway in diabetic complications	VEGFA/SERPINE1/NOX4/MMP2/F3/PIK3CB/PIK3CA/BCL2/CDK4
Nitrogen metabolism	CA12/CA2/CA1/CA3/CA9
Lipid and atherosclerosis	PPARG/RXRA/SRC/BCL2L1/PIK3CB/CYP2C9/PIK3CA/ERN1/MMP3/GSK3B/BCL2/MMP9
Small cell lung cancer	CDK2/RXRA/BCL2L1/PIK3CB/PIK3CA/BCL2/CCNE1/CDK4
Proteoglycans in cancer	VEGFA/ESR1/SRC/KDR/FGFR1/MET/IGF1R/MMP2/PIK3CB/PIK3CA/MMP9
Rap1 signaling pathway	VEGFA/SRC/KIT/KDR/FGFR1/MET/IGF1R/INSR/PIK3CB/PIK3CA/PGF
Chemical carcinogenesis - receptor activation	VEGFA/ESR2/ESR1/CYP1B1/CHRNA7/RXRA/SRC/PIK3CB/CYP3A4/PIK3CA/BCL2
Breast cancer	ESR2/ESR1/KIT/FGFR1/IGF1R/PIK3CB/PIK3CA/GSK3B/CDK4
Cellular senescence	CDK2/CCNB1/SERPINE1/IGFBP3/PIK3CB/PIK3CA/CCNE1/CDK4/CDK1
Focal adhesion	VEGFA/SRC/KDR/MET/IGF1R/PIK3CB/PIK3CA/PGF/GSK3B/BCL2
Regulation of lipolysis in adipocytes	PTGS1/ADORA1/INSR/PTGER3/PIK3CB/PIK3CA
Bladder cancer	VEGFA/SRC/MMP2/MMP9/CDK4

Table S3. Top 20 clustering bar charts of KEGG pathway (Gigantol).

Description	Gene ID
Prostate cancer	IGF1R/CDK2/HSP90AA1/HSP90AB1/ERBB2/MMP3/PDGFRA/BRAF/MAP2K2/NFKB1/CREBBP/PDGFRB/GSTP1/PIK3R1
Endocrine resistance	IGF1R/ESR1/ESR2/RPS6KB1/CDK4/ERBB2/MAPK8/BRAF/NCOR1/MAP2K2/FOS/JUN/PIK3R1
HIF-1 signaling pathway	IGF1R/RPS6KB1/INSR/EGLN1/PRKCA/ERBB2/PRKCG/MAP2K2/NFKB1/SLC2A1/HIF1A/CREBBP/PIK3R1
EGFR tyrosine kinase inhibitor resistance	IGF1R/RPS6KB1/PRKCA/ERBB2/PDGFRA/BRAF/PRKCG/MAP2K2/PDGFRB/AXL/PIK3R1
Chemical carcinogenesis - receptor activation	ESR1/ESR2/RPS6KB1/HSP90AA1/HSP90AB1/PRKCA/PRKCG/MAP2K2/FOS/ADRB2/NFKB1/JUN/KLF5/CHRNB4/CYP3A4/PIK3R1
Glioma	CALM1/IGF1R/CDK4/PRKCA/PDGFRA/BRAF/PRKCG/MAP2K2/PDGFRB/PIK3R1
Fluid shear stress and atherosclerosis	CALM1//MAPK8/FOS/NFKB1/JUN/NFE2L2/NOX1/KEAP1/GSTP1/PIK3R1/PRKCZ
Neurotrophin signaling pathway	CALM1/PSEN2/ABL1/MAPKAPK2/MAPK8/BRAF/MAP2K2/NFKB1/JUN/PSEN1/PTPN11/PIK3R1
Kaposi sarcoma-associated herpesvirus infection	CALM1/PIK3CG/CDK4/PTGS2/MAPKAPK2/MAPK8/SYK/LYN/MAP2K2/FOS/NFKB1/JUN/HIF1A/CREBBP/PIK3R1
Choline metabolism in cancer	RPS6KB1/PRKCA/MAPK8/PDGFRA/PRKCG/MAP2K2/FOS/JUN/HIF1A/PDGFRB/PIK3R1
AGE-RAGE signaling pathway in diabetic complications	CDK4/TGFBR1/NOX4/PRKCA/PIM1/MAPK8/NFKB1/JUN/NOX1/PIK3R1/PRKCZ
PI3K-Akt signaling pathway	IGF1R/PIK3CG/RET/RPS6KB1/CDK2/CDK4/HSP90AA1/INSR/HSP90AB1/PRKCA/ERBB2/EPHA2/SYK/FLT4/PDGFRA/MAP2K2/NFKB1/PDGFRB/ITGB1/PIK3R1
MAPK signaling pathway	IGF1R/RET/INSR/TGFBR1/PRKCA/ERBB2/MAPKAPK2/EPHA2/MAPK8/FLT4/PDGFRA/BRAF/PRKCG/MAP2K2/FOS/NFKB1/JUN/PDGFRB
ErbB signaling pathway	RPS6KB1/ABL1/PRKCA/ERBB2/MAPK8/BRAF/PRKCG/MAP2K2/JUN/PIK3R1
Rap1 signaling pathway	CALM1/IGF1R/INSR/ADORA2B/PRKCA/EPHA2/FLT4/PDGFRA/BRAF/PRKCG/MAP2K2/PDGFRB/ITGB1/PIK3R1/PRKCZ
Renal cell carcinoma	EGLN1/BRAF/MAP2K2/JUN/SLC2A1/HIF1A/PTPN11/CREBBP/PIK3R1
Central carbon metabolism in cancer	RET/ERBB2/PDGFRA/MAP2K2/SLC2A1/HIF1A/PDGFRB/GCK/PIK3R1
Calcium signaling pathway	CALM1/HTR2A/RET/ADORA2B/PRKCA/ERBB2/FLT4/PDGFRA/MYLK/PRKCG/ADRB2/DRD1/P2RX4/PDGFRB/CYSLTR2/TACR1
Chemical carcinogenesis - reactive oxygen species	PTPN1/NOX4/ABL1/MAPK8/BRAF/MAP2K2/FOS/NFKB1/JUN/NFE2L2/HIF1A/NOX1/PTPN11/KEAP1/PIK3R1
Breast cancer	IGF1R/ESR1/ESR2/RPS6KB1/CDK4/ERBB2/FLT4/BRAF/MAP2K2/FOS/JUN/PIK3R1

Table S4. Specific sequences of primers used in RT-qPCR.

Gene	Species		Primer sequence (5' → 3')
NF-kB1	Danio rerio	Forward	AACAGCAGTTGATGGGCCTT
		Reverse	CACCCATAGCGGAACCTGAA
TP53	Danio rerio	Forward	GGCGAACATTTGGAGGGAGA
		Reverse	TGAAGCACCAGCTTGACTGT
BCL2L	Danio rerio	Forward	
		Reverse	
BCL2	Danio rerio	Forward	
		Reverse	
GSK-3β	Danio rerio	Forward	
		Reverse	
SRC	Danio rerio	Forward	
		Reverse	
FOS	Homo sapiens	Forward	
		Reverse	
JUN	Homo sapiens	Forward	
		Reverse	
NF-kB1	Homo sapiens	Forward	
		Reverse	
GAPDH	Homo sapiens	Forward	
		Reverse	
GAPDH	Danio rerio	Forward	
		Reverse	
